# Impact of surgical-site infection on health utility values: a meta-analysis

**DOI:** 10.1093/bjs/znad144

**Published:** 2023-06-12

**Authors:** Agi M McFarland, Sarkis Manoukian, Helen Mason, Jacqui S Reilly

**Affiliations:** Faculty of Health Sciences and Sport, University of Stirling, Stirling, UK; Faculty of Health Sciences and Sport, University of Stirling, Stirling, UK; Glasgow Caledonian University Yunus Centre for Social Business, Glasgow; Faculty of Health Sciences and Sport, University of Stirling, Stirling, UK; Glasgow Caledonian University Yunus Centre for Social Business, Glasgow; Faculty of Health Sciences and Sport, University of Stirling, Stirling, UK; Health and Safeguarding Health through Infection Prevention (SHIP) Research Group, Glasgow

## Abstract

**Background:**

Surgical-site infections (SSIs) are recognized as negatively affecting patient quality of life. No meta-analysis of SSI utility values is available in the literature to inform estimates of this burden and investment decisions in prevention.

**Methods:**

A systematic search of PubMed, MEDLINE, CINAHL, and the National Health Service Economic Evaluation Database was performed in April 2022 in accordance with PROSPERO registration CRD 42021262633. Studies were included where quality-of-life data were gathered from adults undergoing surgery, and such data were presented for those with and without an SSI at similar time points. Two researchers undertook data extraction and quality appraisal independently, with a third as arbiter. Utility values were converted to EuroQol 5D (EQ-5D™) estimates. Meta-analyses were conducted using a random-effects model across all relevant studies, with subgroup analyses on type and timing of the SSI.

**Results:**

In total, 15 studies with 2817 patients met the inclusion criteria. Six studies across seven time points were used in the meta-analysis. The pooled mean difference in EQ-5D™ utility in all studies combined was –0.08 (95 per cent c.i. −0.11 to −0.05; prediction interval −0.16 to −0.01; *I*^2^ = 40 per cent). The mean difference in EQ-5D™ utility associated with deep SSI was −0.10 (95 per cent c.i. −0.14 to −0.06; *I*^2^ = 0 per cent) and the mean difference in EQ-5D™ utility persisted over time.

**Conclusion:**

The present study provides the first synthesized estimate of SSI burden over the short and long term. EQ-5D™ utility estimates for a range of SSIs are essential for infection prevention planning and future economic modelling.

## Teaser

Surgical-site infection (SSI) presents a considerable economic burden and greatly affects the quality of life of patients. This study is the first to synthesize health-related utility estimates of the associated decrement using a meta-analysis of published work. Estimates are provided for overall impact, SSI type, and time.

## Introduction

Healthcare-associated infections (HAIs) are globally the most frequently reported adverse events. Surgical-site infection (SSI) is the most surveyed and frequent HAI in low- and middle-income countries, and the second most frequent HAI in Europe and the USA^1^. Once developed, SSIs present a significant burden in terms of disability and excess duration of hospital stay^2^, and contribute to the development of antimicrobial resistance through the increased use of antimicrobials^3^. Postoperative recovery is extended by SSI and increased use of healthcare resource is required. This impact on morbidity and mortality has a significant effect on patient quality of life^4^.

A recent systematic review^5^ highlighted the negative impact of SSI across six themes of patient functioning, namely physical, psychological, social, spiritual, economic, and the health care worker–patient relationship. Patients who develop SSI after surgery have a longer hospital stay by up to 24 days according to some estimates^6^, with an even greater impact in older people and those infected with resistant microbial strains. Deep SSIs have been reported to be associated with substantial levels of suffering and pain, which may become lifelong^7^. SSIs are also associated with poorer clinical pain and functional outcomes in a variety of surgical categories^8,9^. In an attempt to reduce the burden of such infections, SSI prevention is a mainstay of surgical patient care at both national^4,10^ and international^1^ levels.

Economic evaluations of interventions are used to inform investment decisions in healthcare within a constrained-resource context^11^. Cost–utility analysis, a specific form of cost-effectiveness analysis whereby effectiveness is measured through utility^12^, is ideally suited to explore the economic implications of SSI prevention strategies. Utility can be considered a health valuation on a scale anchored at 0 for dead and 1 for perfect health. This allows for a common outcome unit for interventions that affect both morbidity and mortality^13^, such as SSI. Utility values can subsequently be used to calculate quality-adjusted life years (QALYs), which are the standard health outcome measure sought by the National Institute for Health and Care Excellence for any interventions commissioned for the National Health Service (NHS) and other public sector bodies in the UK^14^. A recent systematic review^15^ identified a paucity of high-quality economic evaluations examining the benefits of SSI prevention. Synthesized utility values for SSI that could be used to generate such evidence are also lacking.

Therefore, a systematic review was undertaken to identify and summarize the utility decrement associated with SSI in adults undergoing surgery reported in the extant evidence base. This information can be used to both quantify the excess burden posed by SSI as well as for future cost–utility analyses evaluating the cost effectiveness of SSI prevention measures. The primary aim was to estimate the impact of any type of superficial, deep, and organ/space infections on the quality of life, as measured by utility score, of adults undergoing surgery.

## Methods

### Inclusion and exclusion criteria

The PICOS (Population, Intervention, Comparison, Outcomes, and Study) framework^16^ was used to guide the inclusion criteria. The population comprised adults (as specified by the trialists) receiving any surgery in any setting. Interventions included any adult with any confirmed SSI using any prespecified diagnostic criteria. Comparators were adults undergoing surgery with no SSI. The main outcome measure was utility as measured by a standardized, validated quality-of-life questionnaire, such as EuroQol 5D (EQ-5D™; EuroQol Group, Rotterdam. The Netherlands) or Short Form 36 (SF-36, Ware, Kosinski and Keller, Boston. USA^®^^17^). Any study in which quality-of-life data were gathered prospectively or retrospectively from adults undergoing surgery, with such data presented for those with and without SSI at similar time points, was included. The main outcome effect measure was mean difference in EQ-5D™ utility. Study designs that were anticipated to fulfil this criterion were RCTs, controlled trials, case–control studies, matched control studies, or economic evaluation or modelling studies that completed primary data collection for utility values. Information was sought directly from authors if missing from published reports.

Studies were excluded if no set diagnostic criteria for SSI were used, secondary quality-of-life data from published sources (for example, economic modelling studies using published utility values that they did not themselves gather) were used, and studies on children. Studies for which the full text was not available (either published, unpublished or directly from the authors) were also excluded.

### Search

The systematic review was registered with PROSPERO (registration number CRD 42021262633). The registration acted as the protocol for this review. Studies that met the PICOS criteria and published in English were included. The following databases were searched from inception to April 2022: PubMed, MEDLINE via EBSCO host, CINAHL via EBSCO host, and the NHS Economic Evaluation Database.

A prespecified search strategy was formulated and adapted for each database’s conventions; details of the main search strategy are available in the *supplementary material*. The NHS Economic Evaluation Database was searched using Medical Subject Headings only. Reference lists in relevant articles were screened to identify any further potential papers.

### Study selection and data extraction

Two authors independently examined the titles and abstracts identified by the search strategy to remove any duplicate records and irrelevant reports. Full-text versions of potentially relevant studies identified by at least one author were retrieved and evaluated. The same two authors independently assessed each study to determine whether it met the eligibility criteria, and extracted data using a standardized data extraction form developed for this review. Any disagreements were resolved by discussion between the authors, with a further author acting as arbiter. The data extraction form included the following: general information (author(s), title, source, contact address, year of study, country of study, year of publication); trial characteristics (design, time horizon, quality-of-life instrument used); participants (baseline characteristics, type of surgery, inclusion and exclusion criteria, sample size, and number of patients allocated to each group or patient cohort details); interventions (SSI type; superficial, deep, organ/space); and outcomes (utility measure in both infected/non-infected groups).

All study outcomes were converted to a single index value of health state utility (EQ-5D™^18^) if not already presented as such in the studies. SF-12^®^ and SF-36^®^ summary measures are not preference-based and as such cannot be used to subsequently calculate QALYs^17^. Conversion to EQ-5D™ values is required, so SF-12^®^ study-level data (mean and standard error) and SF-36^®^ summary measures were converted to EQ-5D™ preference scores using UK tariff values^19^. SF-12^®^ scores were converted using the two-variable model outlined by Lawrence and Fleishman^20^. SF-36^®^ data were converted using Model EQ (1) from Ara and Brazier^21^. Synthesis was planned using a random-effects meta-analysis model, which assumes that the study effect sizes are different and that the collected studies represent a random sample from a larger population of studies. Heterogeneity was explored with forest plots and an χ² test. In addition, the *I*² statistic describing the percentage variation that may be attributed to between-study heterogeneity was calculated. The prediction interval, used to estimate the true effect size and plotting a distribution of true effects^22^, was also calculated. Finally, a funnel plot was generated^23^. Subgroup analysis and meta-regression was planned by infection type according to Centers for Disease Control and Prevention criteria (superficial, deep, organ/space), wound classification (clean, clean contaminated, contaminated, dirty or infected)^24^, National Nosocomial Infections Surveillance risk index criteria^25^, and time after surgery if possible. Analyses were undertaken using Stata^®^ statistical software^26^ and the Prediction Intervals Program^27^.

### Quality assessment

Study quality assessment was completed independently by two authors using the STROBE checklist for case–control studies^28^, the Drummond checklist for economic evaluations^29^, and the Cochrane Risk of Bias (RoB 2) tool^16^ for RCTs. RCTs in which randomization of the intervention was not relevant to the SSI outcomes of primary interest of the present review were also assessed using the STROBE checklist. Study components were rated using the relevant ratings in each instrument. Utility valuation studies were rated against the components presented by Stalmeier *et al.*^30^. Any disagreement was resolved through discussion. Quality assessment was evaluated both within and across all included study types.

## Results

### Study selection and characteristics

**Fig. 1:**
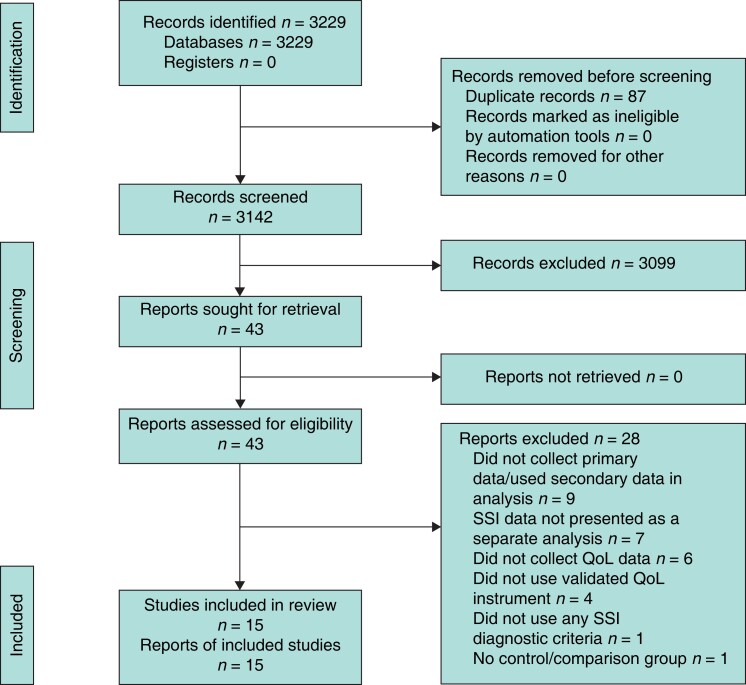
**PRISMA flow diagram showing selection of articles for review**SSI, surgical-site infection; QoL, quality of life.

The search identified 3142 titles and abstracts after removal of duplicates. A total of 43 articles were initially identified as having potential and full texts of these were retrieved. Seven articles reported SSI utility data included with the main study comparison groups. The authors were contacted directly for information, but none was obtained. These articles were subsequently excluded. A further 21 were excluded for other reasons and 15 studies met the inclusion criteria (*Fig. 1*). Characteristics of all included studies are summarized in *Table S1*.

Publication dates ranged from 2002 to 2021, with a peak in publication rates in the years 2018–2019. The majority of the studies were set in the USA^31–36^, then the UK^37–39^, and Australia^40,41^, with single studies from Brazil^42^, Spain^43^, Denmark^44^ or a combination of European countries^8^. Most studies examined SSI in orthopaedic surgery involving the spine^8,31–33,35,42^, total joint replacement^37,40,41,43^, trauma^38^ or a combination^36^. Single studies focused on obstetric^44^ or non-obstetric^34^ and vascular^39^ procedures. The studies predominantly focused on deep^8,31–35,38,42^ and organ/space^40,41^ SSI. Only one study^43^ looked at superficial infections and four^36,37,39,44^ included all infection types. All studies used a case–control design, with the exception of two RCTs^38,39^, an economic evaluation^44^, and a health state valuation using time trade-off interviews^37^. All studies used standardized validated quality-of-life questionnaires: EQ-5D™^31,33,38,39,44^, SF-36^®8,32,35,36,41–43^ or SF-12^®34,40^. Eight^8,34–36,40–43^ studies required utility conversions to EQ-5D™.

### Quality assessment

The single economic evaluation^44^ and health state valuation study^37^ completed most criteria on each respective quality assessment tool^29,30^. One RCT^39^ was assessed as having a low risk of bias, whereas the other^38^ had some concerns, mainly owing to elements in domain 2 (risk of bias due to deviations from the intended interventions) of the Cochrane RoB 2 tool^45^. The case–control studies were of reasonable quality. Generally, criteria relating to the title and abstract, introduction, and initial methods of the STROBE checklist^28^ were completed well. There was variability in checklist elements related to bias and statistical methods. Only two studies^8,36^ provided a flow diagram. One^42^ did not address limitations, and another^36^ did not provide details of funding. All other studies performed well on checklist criteria relating to discussion and other information. Overall, the quality of the included studies was fair, with case–control methodology being associated with lower levels of reporting quality than economic evaluation and health state valuation. Results of the quality assessments for all included studies can be found in the *Table S2* (case control studies), *Table S3* (RCTs), *Table S4* (economic evaluation), and *Table S5* (health state valuation).

### Study-level utility impact of surgical-site infection

Single-study estimates of the utility decrement associated with SSI ranged from –0.203 in deep SSI following total joint replacement (34 cases of SSI) at more than 1 year after surgery^41^ to –0.03 in all SSIs requiring antibiotic treatment 30 days after caesarean section (39 cases of SSI) using standard wound dressing^44^. *Table S6* outlines the study-level estimates of utility associated within the range of SSIs reported at various time points and SSI types.

### Meta-analyses

**Fig. 2:**
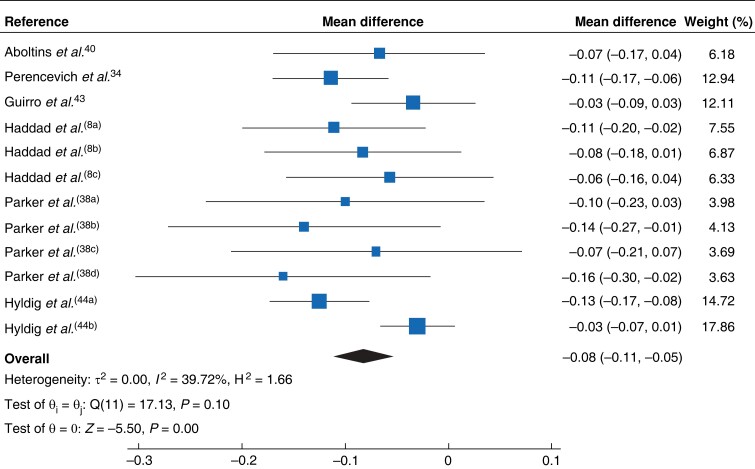
Main meta-analysis outcome measure showing mean difference in EQ-5D™ utility estimates across six included studies Mean differences are shown with 95% confidence intervals. A random-effects model was used for meta-analysis. ^a-d^signify different time points within the same study or for Hyldig the two dressing comparator groups (i.e both arms of the RCT).

The authors of studies that did not report the standard deviation, standard error or confidence interval of mean estimates for quality-of-life scores were contacted directly by e-mail for missing data. One supplied the information, one was no longer available, and no response was received from the others. Thus, data from six studies^8,34,38,40,43,44^ across seven time points (1 to more than 12 months) and a range of procedures (arthroplasty, spinal, lower limb trauma, caesarean section, and all non-obstetric in 1 study site) were available for meta-analysis. The mean difference in EQ-5D™ utility in all studies combined was –0.08 (95 per cent c.i. −0.11 to −0.05; *I*^2^ = 40 per cent) (*Fig. 2*).

#### Subgroup analyses

**Fig. 3:**
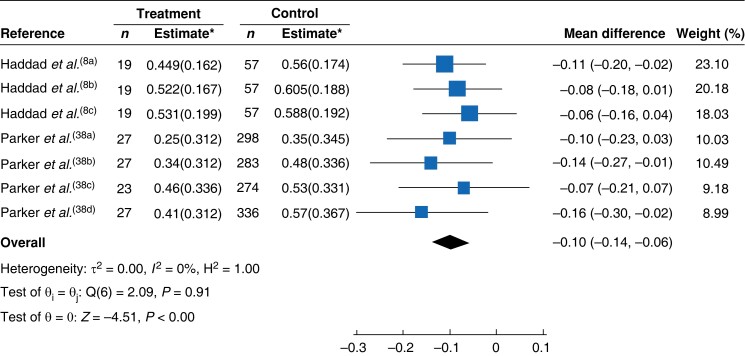
Forest plot for EQ-5D™ mean difference in deep surgical-site infection only Mean differences are shown with 95% confidence intervals. *Values are mean(s.d.). EQ-5D™ utility estimates. A random-effects REML model was used for meta-analysis. ^a-d^signify different time points within the same study or for Hyldig the two dressing comparator groups (i.e both arms of the RCT).

Data from two studies^8,38^ across five time points were available for meta-analysis of deep SSI only. The mean difference in EQ-5D™ utility associated with deep SSI was −0.10 (95 per cent c.i. −0.14 to −0.06; *I*^2^ = 0 per cent) (*Fig. 3*).

**Fig. 4:**
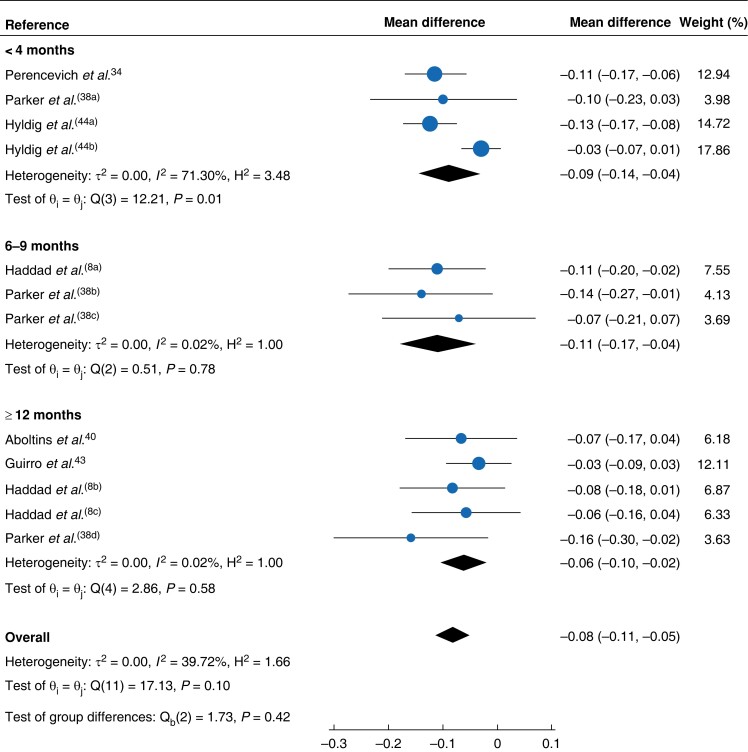
Forest plot for EQ-5D™ mean difference across all surgical-site infection types at different time points Mean differences are shown with 95% confidence intervals. A random-effects REML model was used for meta-analysis. ^a-d^signify different time points within the same study or for Hyldig the two dressing comparator groups (i.e both arms of the RCT).

To explore the impact of time since surgery, eligible studies were regrouped into three time-point categories of less than 4 months^34,38,44^, 6–9 months^8,38^, and 12 months or more^8,38,40,43^. The mean difference in EQ-5D™ utility was −0.09 (−0.14 to −0.04; *I*^2^ = 71 per cent) at less than 4 months, −0.11 (−0.17 to −0.04; *I*^2^ = 0.02 per cent) at 6–9 months, and −0.06 (−0.10 to −0.02; *I*^2^ = 0.02 per cent) at 12 months or more (*Fig. 4*). No data were available for the other planned subgroup analyses.

### Heterogeneity

**Fig. 5:**
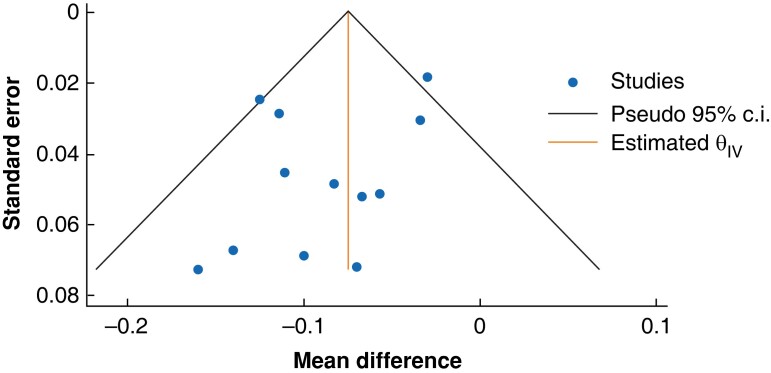
Funnel plot of all included studies

The estimate of mean difference in EQ-5D™ utility across all studies may represent moderate heterogeneity with an *I*^2^ value of 40 per cent^16^. An asymmetric funnel plot, as evidenced by scatter points outwith the pseudo 95 per cent confidence interval either side of the mean summary effect estimate^46^, may indicate this is due to publication bias (*Fig. 5*). Lower *I*^2^ values from subgroup analyses in which only one type of SSI was considered (for example *I*^2^ = 0 per cent for deep SSI) and higher ones when different types of surgery were considered (for example *I*^2^ = 71 per cent for all SSI types at less than 4 months since surgery) suggest a clinical source of the overall moderate heterogeneity found in the main meta-analysis outcome measure. For the main outcome measure, the true effect size in 95 per cent of all comparable populations fell in the interval −0.16 to −0.01 (Figure S6).

## Discussion

The primary aim of the review was to estimate the impact of an SSI on quality of life, as measured by utility score, for adults undergoing surgery of any type for all SSIs.

In the meta-analysis of six studies across all time points since surgery, the mean difference in EQ-5D™ utility across all SSI types was −0.08 (95 per cent c.i. −0.11 to −0.05; *I*^2^ = 40 per cent) (*Fig. 2*). For deep SSI, the utility decrement associated with these infections dropped to −0.10 (−0.14 to −0.06; *I*^2^ = 0 per cent). The greatest utility decrement was associated with the interval 6–9 months after surgery (−0.11, −0.17 to −0.04; *I*^2^ = 0.02 per cent) and this reduced with time (−0.06, −0.10 to −0.02; *I*^2^ = 0.02 per cent, at 12 months or more).

The 0.08 utility decrement in the primary outcome meta-analysis may appear relatively minimal. There are, however, certain contextual and methodological considerations to acknowledge. Analysing beyond the EQ-5D™ overall mean score provides insight into which aspects of health are contributing to the reported changes^47^. Thus, a 0.08 drop in overall EQ-5D™ utility may represent changes in up to two health state dimensions of the valuation instrument^48^. In practical terms, this could mean that SSIs have influenced health to the extent that moderate, severe or even extreme problems are now present in mobility, self-care, usual activities, pain/discomfort or anxiety/depression. Indeed, given the prediction interval from the analysis, some patients may experience decrements of up to 0.16, compounding this effect. The distribution of true effects indicates that 95 per cent of patients in all comparable populations would consistently experience a decrement in utility due to SSI, highlighting the importance of this clinical issue. The utility value decrement must also be viewed in the context of the high global prevalence of SSI^1^, resulting in significant cumulative effects on health at the population level. The finding that this decrement is present even at 12 months or more after surgery further highlights the importance of prevention efforts and investment in this area.

To the authors’ knowledge, this is the first systematic review to combine utility valuations of SSI in a meta-analysis. Previous work^49^ that aimed to summarize the evidence base on SSI utility values identified 28 studies, but no meta-analysis was completed. Additionally, only nine of these studies used patient-level data for health state valuations. The remaining 19 used values mainly sourced from the literature and were of poor methodological quality. The range of utility decrement reported^49^ is comparable at the lower end of estimates (0.04 versus 0.03 in this study) but not at high the higher end (0.48 versus 0.203 in this study). Interestingly, the authors concluded that the SSI utility decrement is suggested to be near 0.1, in alignment with the findings from the meta-analysis presented here, with an overall mean difference in EQ-5D™ utility of 0.08 in all studies combined.

The scope for heterogeneity across studies examining SSI is wide because of the diverse nature of the infection, evidenced by multilevel diagnostic criteria, surgical classifications^24^, and specialties. The lack of previous meta-analyses in this field has been justified on this basis^50^. The included studies for this review predominantly examined deep SSI in orthopaedic surgery and so meta-analysis was deemed appropriate. Studies were not excluded based on surgery and infection type, in line with the primary aim of the review. The moderate levels of heterogeneity found in the main meta-analysis outcome measure support this decision.

Overall, the methodological quality of the studies in this review was good. Several studies did not, however, report the standard deviation, standard error or confidence interval for mean estimates of quality-of-life scores, and only one author responded to the request for such information. Thus, the scope for meta-analysis was limited by the reporting in the original studies. This highlights the need for consistency in reporting of future work in this field, such as the use of reporting tools or checklists for peer review. A further limitation of this review is the lack of data relating to superficial and organ/space SSI and surgical classifications. Although the orthopaedic focus of most included studies facilitated meta-analysis, the utility decrements reported are heavily weighted towards this surgical specialty and may not be appropriate for use in other surgical settings.

Global guidelines^1^ on the prevention of SSI from the WHO specifically call for robust SSI economic and burden studies to address clinical need. The present study contributes to this call. As with European SSI surveillance, where specific surgical categories are used as indicator metrics for overall SSI burden^51^, the present findings may offer insight into a wider scope of surgical specialty than those of the studies in the meta-analyses (orthopaedic and obstetrics). Given the paucity of synthesized estimates of SSI impact on health-related quality of life, the review is presented as a reference point from which clinicians may begin to infer utility decrements within wider specialty groupings based on associated clinical risk of other surgical categories. A synthesized estimate offers methodological advantage and increased precision over those derived from single studies^52^. As such, the present review provides a higher rank of evidence^53^ for this purpose than previously published work.

The findings of this study confirm the significant burden that SSIs present to the surgical patient population both in the short and long term. The authors sought to quantify the known clinical burden of these infections and demonstrate the long-term (12 months after surgery and beyond) impact on patient quality of life. EQ-5D™ utility estimates for a range of SSIs for use in future economic modelling are also provided. Recommendations for future work in this area include improvement in the quality and consistency of reporting quality-of-life outcomes and data collection across the full scope of the SSI clinical pathway and timeline.

## Supplementary Material

znad144_Supplementary_Data

## Data Availability

Data generated for this review are available directly from the corresponding author.
