# Supplementary material for: Impact of surgical-site infection on health utility values: a meta-analysis
Source: Br J Surg. 2023 Jun 12;110(8):942–9. doi: 10.1093/bjs/znad144 (PMC10361680; doi:10.1093/bjs/znad144)
Supplement: znad144_Supplementary_Data [file znad144_supplementary_data.zip › Supplementary_Material.docx]

**Title:** The impact of surgical site infection on health utility values: meta-analysis

**Full name of each author:**

Agi M McFarland^1^

Sarkis Manoukian^2^

Helen Mason^2^

Jacqui S Reilly^3^

**The department(s) and institution(s) to which the work should be attributed:**

^1^University of Stirling Faculty of Health Sciences and Sport

^2^Glasgow Caledonian University Yunus Centre for Social Business and Health

^3^Glasgow Caledonian University Safeguarding Health through Infection Prevention (SHIP) Research Group.

**Author responsible for correspondence:**

Agi, McFarland

Faculty of Health Sciences and Sport

University of Stirling

Stirling

FK9 4LA

Tel: +44 1786 473 171

Email: [agi.mcfarland@stir.ac.uk](mailto:agi.mcfarland@stir.ac.uk)

ORCID ID: <https://orcid.org/0000-0002-5061-273X>

**Supplementary Materials - Index**

| **Supplementary Methods** |  |
| --- | --- |
| Main search strategy | *pag. 2* |
| **Supplementary Figures and Tables** |  |
| Table S1: Overview of Included Studies | *pag. 3* |
| Table S6: Study level utility impact of SSI | *pag. 5* |
| Figure S1: Distribution of true effects | *pag. 7* |
| Table S2: Quality assessment of all included case control studies | *pag. 8* |
| Table S3: Quality assessment of included RCTs | *pag. 47* |
| Table S4: Quality assessment of economic evaluation | *pag. 60* |
| Table S5: Quality assessment of health state valuation | *pag. 63* |
| **References** | *pag. 67* |
|  |  |

**Supplementary Methods**

Main search strategy

| 1: Combine with OR: |
| --- |
| economics  economic*  cost  direct cost*  economic eval*  economic analys*  cost utili*  cost effect*  cost benefit* |
| 2: Combine with OR: |
| Eq 5d  Eq-5d  Eq-5d*  Eq5d*  Euroqol  General health questionnaire  Ghq  Health utility*  Hql  Hrql  Hrqol  Hui  Hye  Life quality  NHP  Nottingham health profile  Psychological general well*  Qaly  Qol  Quality adjusted life year  Quality of life  Quality of well*  Qwb  Short form OR Short Form-36 Health Survey (SF-36) MH  Shortform |
| 3: Combine with OR: |
| Surgical Wound Infection MH  surgical site infection*  SSI  Surgical wound infection  SWI |
| Combine 1 and 2 |
| Combine (1+2) AND 3 |

**Supplementary Figures and Tables**

**TABLE S1:** Overview of Included Studies

| **Study reference** | **Setting** | **Study design** | **No. of cases (n)** | **No. of controls (n)** | **SSI type** | **Valuation instrument** | **Timing of outcome assessment** |
| --- | --- | --- | --- | --- | --- | --- | --- |
| Aboltins (1) | Early prosthetic joint infection in THA, Australia. | Case control | 19 | 79 | CDC definition of organ/space SSI | SF-12 | Baseline: 2 – 8 weeks pre op  Outcome: 12 months post operatively |
| Cahill(2) | Total joint replacement (THA or TKA) complicated by deep infection, Australia. | Case control | 34 | 62 | No stated but treatment options listed as “antibiotics”, “washout/antibiotics”, “2-stage revision” (p59) | SF-36 | Measured once. Mean follow up 98 months controls, 57 months cases |
| Falavigna (3) | Deep wound infection in spinal surgery, Brazil. | Case control | 13 | 39 | “positive cultures from subfascial fluid” (p400) | SF-36 | Pre-op score and post op score with “median follow up duration of 22 months” (p401) |
| Guirro (4) | Superficial wound infection in TKA, Spain. | Case control | 45 | 629 | CDC definition of superficial SSI | SF-36 | Baseline: “preoperative” but no timeline given  Outcome: “final follow up”; mean length of follow up for all study participants 74.57 months (SD ± 7.1) |
| Haddad(5) | Deep SSI in adult spinal deformity surgery, Europe^[[1]](#footnote-1)^ | Case control | 20 | 60 | CDC definition of deep SSI | SF-36 | Baseline: “preoperatively” but no timeline given  Outcome: assessed at 6, 12 and 24 months post operatively |
| Hyldig (6) | SSI post CS in obese (BMI ≥ 30 kg/m^2^) women, Denmark. | Economic evaluation | 20 (iNPWT group)  39 (standard dressing group) | 410 (iNPWT group)  417 (standard dressing group) | “infection at the surgical site requiring antibiotic treatment with the first 30 days after CS” (p620) | EQ-5D-5L  Danish cross walk index | 30 days post CS |
| Kuhns(7) | Deep SSI post subaxial dorsal cervical fusion, USA. | Case control | 14 | 21 | CDC definition of deep SSI | EQ-5D | Pre operatively, 6 months and 12 months |
| Matza (8) | Staphylococcus SSI post TKA/THA and spinal surgery  Vignettes on general population (n = 201), UK. | Health state valuation | N/A | N/A | “superficial”, “deep infection followed by DAIR”, “deep infection (two-stage revision arthroplasty)”, “infection not requiring surgery” “infection requiring surgery” | Vignettes and TTO | “overall utility decrease during a 1-year period in which the SSI occurs” (p825) |
| Mok (9) | Deep SSI following instrumented posterior spinal fusion, USA. | Case control | 16 | 16 | “deep wound infection requiring treatment by operative irrigation and debridement, positive intraoperative cultures from sub fascial intraoperative specimen, minimum 2 year clinical follow up, and radiographic follow up at greater than 1 year after surgery” (p579) | SF-36 v2.0 Physical component scores only | “most recent follow up visit or by mail” (p579) |
| Parker (10) | Participants from WOLLF study with open fractures of the lower limb by ortho/plastics, UK. | RCT | 35 | 423 | CDC definition of deep SSI | EQ-5D-3L with Dolan UK algorithm and SF-6D | Baseline, 3, 6, 9 and 12 months post randomisation |
| Pennington(11) | Deep SSI following posterior lumber decompression and fusion, USA. | Case control | 18 | 18 | CDC definition of deep SSI | EQ-5D | Pre operatively and 6 months post op for controls and 6 months post debridement for SSI |
| Perencevich(12) | SSI following non obstetric inpatient or outpatient operating room procedure, USA. | Case control | 50 | 123 | NNIS surveillance criteria at 30 days | SF-12 | 8 weeks after surgery and 4 weeks before surgery. Pre-operative SF-12 based on recall |
| Petilon (13) | Deep SSI following lumber fusion, USA. | Case control | 30 | 30 | CDC definition of deep SSI | SF-36 | Pre operatively and 2 years post operatively |
| Totty(14) | SSI following clean or clean contaminated vascular surgery, UK. | RCT | 18 | 73 | ASEPSIS score or CDC criteria | EQ-5D-3L | Baseline, day 7 and day 30 |
| Whitehouse (15) | SSI following orthopaedic surgery, USA. | Case control | 23 | 23 | CDC definition of superficial incisional, deep incisional and organ/space SSI  (did not report these separately) | SF-36 | “approximately 1 year after” detection of SSI or after surgery |

THA: total hip arthroplasty

TKA: total knee arthroplasty

CS: caesarean section

BMI: body mass index

iNPWT: incisional negative pressure wound therapy

WOLLF: Wound in Open Lower Limb Fracture

RCT: randomised controlled trial

**TABLE S6:** Study level utility impact of SSI

| Timing (post op.) | *30 days* | | *8 weeks* | | *3 months* | | *6 months* | | *9 months* | | *12 months* | | *≤12 months* | |
| --- | --- | --- | --- | --- | --- | --- | --- | --- | --- | --- | --- | --- | --- | --- |
| SSI Type |  | |  | |  | |  | |  | |  | |  | |
|  | SSI | No SSI | SSI | No SSI | SSI | No SSI | SSI | No SSI | SSI | No SSI | SSI | No SSI | SSI | No SSI |
| **Superficial** |  |  |  |  |  |  |  |  |  |  |  |  |  |  |
| Matza (8) |  |  |  |  |  |  |  |  |  |  | 0.76 (0.24) |  |  |  |
| Guirro (4)* |  |  |  |  |  |  |  |  |  |  |  |  | 0.610 (0.211) | 0.644 (0.197) |
| **Deep** |  |  |  |  |  |  |  |  |  |  |  |  |  |  |
| Cahill (2)* |  |  |  |  |  |  |  |  |  |  |  |  | 0.489 | 0.692 |
| Falavigna (3)* |  |  |  |  |  |  |  |  |  |  |  |  | 0.581 | 0.711 |
| Haddad(5)* |  |  |  |  |  |  | 0.449 (0.162) | 0.560 (0.174) |  |  | 0.522 (0.167) | 0.605 (0.188) | 0.531 (0.199) | 0.588 (0.192) |
| Kuhns (7) |  |  |  |  |  |  | 0.54 | 0.69 |  |  | 0.68 | 0.63 |  |  |
| Matza(8) |  |  |  |  |  |  |  |  |  |  | 0.61 (0.24)  0.47 (0.37) |  |  |  |
| (9) |  |  |  |  |  |  |  |  |  |  |  |  |  |  |
| Parker (10)^ |  |  |  |  | 0.25 (0.06) | 0.35 (0.02) | 0.34 (0.06) | 0.48 (0.02) | 0.46 (0.07) | 0.53 (0.02) | 0.41 (0.06) | 0.57 (0.02) |  |  |
| Pennington (11) |  |  |  |  |  |  | 0.56 | 0.67 |  |  |  |  |  |  |
| Petilon (13)* |  |  |  |  |  |  |  |  |  |  |  |  | 0.416 | 0.479 |
| Whitehouse(15)* |  |  |  |  |  |  |  |  |  |  | 0.511 | 0.614 |  |  |
| **Organ/space** |  |  |  |  |  |  |  |  |  |  |  |  |  |  |
| Aboltins (1)* |  |  |  |  |  |  |  |  |  |  | 0.595 (0.192) | 0.662 (0.204) |  |  |
| **All SSI** |  |  |  |  |  |  |  |  |  |  |  |  |  |  |
| Hyldig (6)(int.group) | 0.738 (0.695 - 781) | 0.863 (0.852 - 0.873) |  |  |  |  |  |  |  |  |  |  |  |  |
| Hyldig (6)(control group) | 0.825 (0.79 - 0.861) | 0.855 (0.845 - 0.866) |  |  |  |  |  |  |  |  |  |  |  |  |
| Totty(14) | 0.51 | 0.68 |  |  |  |  |  |  |  |  |  |  |  |  |
| Perencevich (12)* |  |  | 0.567 (0.182) | 0.681 (0.165) |  |  |  |  |  |  |  |  |  |  |

Cases denotes patients with confirmed SSI, Controls denotes patients with no confirmed SSI within the same study.

Matza et al (2019) was a health state valuation study therefore no Control group data were available.

Mok et al (2009) only provided SF-6 Physical domain scores so EQ-5D conversion was not possible.

All data reported in mean utility as measured by EQ-5D preference scores and 95% CI or SD (where available).

^mean (SE)

*denotes where utility scores were derived from conversion as described in Methods

**FIGURE S1:** Distribution of true effects

**
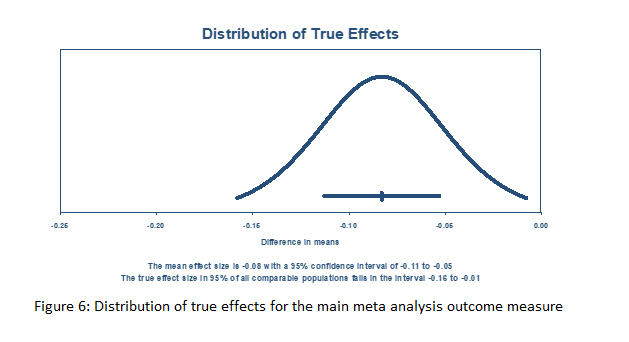
**

**TABLE S2:** Quality assessment of all included case control studies

| STUDY REFERENCE | |  |
| --- | --- | --- |
| ***Aboltins 2013*** | Enter Yes/No/Not clear or NA | Text that helped you make the decision, including page number |
| Title and abstract | | |
| 1a: Indicate the study’s design with a commonly used term in the title or the abstract | Yes | “Nineteen patients treated for PJI after hip arthroplasty with debridement, prosthesis retention and biofilm-active antibiotics were matched to 76 controls who underwent hip arthroplasty with no infection” (p810). |
| 1b: Provide in the abstract an informative and balanced summary of what was done and what was found | Yes | Setting, methods, participants and overview of findings provided in Abstract. |
| Introduction | | |
| 2: Background/rationale  Explain the scientific background and rationale for the investigation being reported | Yes | Section “Introduction” (p810). |
| 3: Objectives  State specific objectives, including any prespecified hypotheses | Yes | “to describe rates of successful infection treatment for patients undergoing treatment for PJI with surgical debridement, prosthesis retention and biofilm-active antibiotics and compare their functional outcomes, QOL and complication rates to patients without PJI” (p810). |
| Methods | | |
| 4: Study design  Present key elements of study design early in the paper | Yes | “case-control study performed at a single tertiary institution” (p811). |
| 5: Setting  Describe the setting, locations, and relevant dates, including periods of recruitment, exposure, follow-up, and data collection | Yes | “St Vincent’s Hospital in Melbourne, Australia”  “consecutive patients undergoing primary hip arthroplasty at SVHM between January 2006 and December 2009”  “Baseline HHS and SF-12 assessments were administered 2-8 weeks prior to hip arthroplasty, and outcome assessments were performed 12 months post arthroplasty” (p811). |
| Participants  6a: Give the eligibility criteria, and the sources and methods of case ascertainment and control selection. Give the rationale for the choice of cases and controls | Yes | “eligible cases were patients who developed PJI some time prior to the 12 month post arthroplasty assessment and had treatment with prosthesis debridement and retention”  “Controls were patients who did not develop PJI within 12 months from arthroplasty” (p811). |
| 6b: For matched studies, give matching criteria and the number of controls per case | Yes | “matched at a ratio of 4 to 1 to cases by age and pre-arthroplasty Harris Hip Score” (p811). |
| 7: Variables  Clearly define all outcomes, exposures, predictors, potential confounders, and effect modifiers. Give diagnostic criteria, if applicable | Yes | QoL outcome measures included HHS and SF-12.  Infection used CDC SSI diagnostic criteria.  Potential confounders were preoperative functional and QoL score, gender, BMI, age adjusted CCI^[[2]](#footnote-2)^, smoking status, previous hip arthroplasty, operation duration, cemented prosthesis, superficial wound complications and presence of PJI. |
| 8: Data sources/measurement  For each variable of interest, give sources of data and details of methods of assessment (measurement). Describe comparability of assessment methods if there is more than one group | Yes | QoL outcome measures included HHS and SF-12. |
| 9: Bias  Describe any efforts to address potential sources of bias | Yes | Protocolised treatment of PJI.  Use of retrospective data; although some prospective data collection also completed.  Sample size relatively small for cases (n = 19). |
| 10: Study size  Explain how the study size was arrived at | No | No information provided on study sample size selection. Selection appears to have been over a set time period but no rationale is provided. |
| 11: Quantitative variables  Explain how quantitative variables were handled in the analyses. If applicable, describe which groupings were chosen and why | Not clear | Tabular data provide counts of cases and controls but only for SF12 data |
| Statistical methods  12a: Describe all statistical methods, including those used to control for confounding | Yes | Section “Statistical analysis” (p811-2) |
| 12b: Describe any methods used to examine subgroups and interactions | NA | No subgroup analysis completed |
| 12c: Explain how missing data were addressed | NA | No missing data |
| 12d: If applicable, explain how loss to follow up was addressed | No | “loss to follow up (16 patients)” p811  No information is provided on how this was addressed in the analysis. |
| 12d: If applicable, explain how matching of cases and controls was addressed | Not clear | “matched at a ratio of 4 to 1 to cases by age and pre-arthroplasty Harris Hip Score” (p811)  No further information provided on matching. |
| 12e: Describe any sensitivity analyses | NA | No sensitivity analysis completed |
| Results | | |
| Participants  13a: Report numbers of individuals at each stage of study— e.g. numbers potentially eligible, examined for eligibility, confirmed eligible, included in the study, completing follow-up, and analysed | Yes | “981 hip arthroplasty operations were performed and minimum 12-month follow-up information was available on 952, with the remaining 59 unavailable…”(p811). |
| 13b: Give reasons for non-participation at each stage | Yes | “…because of death from causes unrelated to surgery (13 patients) or loss to follow up (16 patients”) p811. |
| 13c: Consider use of a flow diagram | No | Not provided. |
| Descriptive data  14a: Give characteristics of study participants (eg demographic, clinical, social) and information on exposures and potential confounders | Yes | Table 1, p812. |
| 14b: Indicate number of participants with missing data for each variable of interest | NA | No missing data |
| 15: Outcome data  Report numbers in each exposure category, or summary measures of exposure | Yes | Tables 3 and 4, p813-4. |
| Main results  16a: Give unadjusted estimates and, if applicable, confounder-adjusted estimates and their precision (eg, 95% confidence interval). Make clear which confounders were adjusted for and why they were included | Yes | Tables 3 and 4, p813-4.  Narrative in section Results (p812-4). |
| 16b: Report category boundaries when continuous variables were categorized | NA | Variables were not categorised |
| 16c: If relevant, consider translating estimates of relative risk into absolute risk for a meaningful time period | No | Not provided. |
| 17: Other analyses  Report other analyses done—e.g. analyses of subgroups and interactions, and sensitivity analyses | Yes | Analysis also examined infection outcomes of cases and complication rates. |
| Discussion | | |
| 18: Key results  Summarise key results with reference to study objectives | Yes | Section Discussion (p814) |
| 19: Limitations  Discuss limitations of the study, taking into account sources of potential bias or imprecision. Discuss both direction and magnitude of any potential bias | Yes | Acknowledges results cannot be generalised to all PJIs treated with debridement and retention, retrospective study design and small sample size of cases (n = 9). |
| 20: Interpretation  Give a cautious overall interpretation of results considering objectives, limitations, multiplicity of analyses, results from similar studies, and other relevant evidence | Yes | Section Discussion p814 – 5. |
| 21: Generalisability  Discuss the generalisability (external validity) of the study results | Yes | “patients treated for early PJI with debridement, prosthesis retiention and biofilm-active antibiotic results in not only successful treatment of infection but also significant improvements in functional and QOL outcomes”  “…cannot be generalised to all PJIs treated with debridement and retention. Each case patient exhibited features that have been shown previously to be associated with good treatment success rates” (p815). |
| Other information | | |
| 22: Funding  Give the source of funding and the role of the funders for the present study and, if applicable, for the original study on which the present article is based | Yes | 2^nd^ author holds an NHMRC Early Career Australian Clinical Fellowship. |

|  | |  |
| --- | --- | --- |
| ***Cahill 2008*** | Enter Yes/No/Not clear or NA | Text that helped you make the decision, including page number |
| Title and abstract | | |
| 1a: Indicate the study’s design with a commonly used term in the title or the abstract | Not clear | “62 uncomplicated TJRs and 34 TJRs complicated with deep infection were compared” (p58). No specific mention of study design. |
| 1b: Provide in the abstract an informative and balanced summary of what was done and what was found | Yes | Abstract sections Methods and Results (p58). |
| Introduction | | |
| 2: Background/rationale  Explain the scientific background and rationale for the investigation being reported | Yes | Section Introduction, p58-9. |
| 3: Objectives  State specific objectives, including any prespecified hypotheses | Yes | “aimed to compare the outcomes of patients with or without infection after TJR. We hypothesised that health related quality of life and disease specific outcomes would be significantly reduced in patients with infection” (p59). |
| Methods | | |
| 4: Study design  Present key elements of study design early in the paper | Yes | Section Materials and Methods (p59). |
| 5: Setting  Describe the setting, locations, and relevant dates, including periods of recruitment, exposure, follow-up, and data collection | Yes | “uncomplicated…patients enrolled in the Australian Care Continuum and Health Outcomes Project between May 1995 and May 1996 who underwent TKR or THR”  “complicated…from an in-patient cost study conducted between July 1995 and July 2001…from the clinical practice of local orthopaedic surgeons” (p59)  Data collection was via questionnaires, either with telephone or face to face interviews or self-completion. |
| Participants  6a: Give the eligibility criteria, and the sources and methods of case ascertainment and control selection. Give the rationale for the choice of cases and controls | Yes | As above and also “Treatment options for the 34 patients complicated with infection included antibiotics (n = 6), washout/antibiotics (n = 8), 2-stage revision (n=15), and excision (n=5)” (p59). |
| 6b: For matched studies, give matching criteria and the number of controls per case | NA | Not a matching study. |
| 7: Variables  Clearly define all outcomes, exposures, predictors, potential confounders, and effect modifiers. Give diagnostic criteria, if applicable | Yes | “Outcomes following TJR were measured using the visual analogue scale for satisfaction, the Western Ontario and McMaster Universities Osteoarthritis Index (WOMAC), the Assessment of Quality of Life (AQoL), and the Short Form 36 (SF-36)” (p59). |
| 8: Data sources/measurement  For each variable of interest, give sources of data and details of methods of assessment (measurement). Describe comparability of assessment methods if there is more than one group | Yes | “sent a package containing the questionnaires, information sheet, and consent forms. Five patients were interviewed by telehpne, 80 were interviewed face to face, and 11 completed the questionnaires without assistance” (p59). |
| 9: Bias  Describe any efforts to address potential sources of bias | Not clear | Normative SF-36 data for 65 – 74 year age groups used due to age of sample size.  Large number of patients declined to participate and also were lost to follow up; no information provided as to how these biases were dealt with. |
| 10: Study size  Explain how the study size was arrived at | Not clear | Inference is study recruited to date limit, not sample size. |
| 11: Quantitative variables  Explain how quantitative variables were handled in the analyses. If applicable, describe which groupings were chosen and why | No | Tabular and graphical data do not provide counts of cases and controls. |
| Statistical methods  12a: Describe all statistical methods, including those used to control for confounding | Yes | Section beginning “Statistical analysis were performed…” to “…the inpatient group it was 0.43 (SD, 0.30)” (p60). |
| 12b: Describe any methods used to examine subgroups and interactions | Yes | “Multiple regression analysis was used” (p60). |
| 12c: Explain how missing data were addressed | Yes | “Missing data were completed by telephone interview” (p59). |
| 12d: If applicable, explain how loss to follow up was addressed | No | “were lost to follow up (12/118)” (p63)  No information is provided on how this was addressed in the analysis. |
| 12d: If applicable, explain how matching of cases and controls was addressed | NA | Not a matching study. |
| 12e: Describe any sensitivity analyses | NA | No sensitivity analysis completed. |
| Results | | |
| Participants  13a: Report numbers of individuals at each stage of study— e.g. numbers potentially eligible, examined for eligibility, confirmed eligible, included in the study, completing follow-up, and analysed | Yes | Section in Materials and Methods beginning “Of these 188 patients…” and ending “…and excision (n = 5)” (p59). |
| 13b: Give reasons for non-participation at each stage | Yes | As above. |
| 13c: Consider use of a flow diagram | No | Not provided. |
| Descriptive data  14a: Give characteristics of study participants (eg demographic, clinical, social) and information on exposures and potential confounders | Yes | Table 1, p59. |
| 14b: Indicate number of participants with missing data for each variable of interest | Yes | Section in Materials and Methods beginning “Of these 188 patients…” and ending “…and excision (n = 5)” (p59). |
| 15: Outcome data  Report numbers in each exposure category, or summary measures of exposure | Yes | Table 1, p59. |
| Main results  16a: Give unadjusted estimates and, if applicable, confounder-adjusted estimates and their precision (eg, 95% confidence interval). Make clear which confounders were adjusted for and why they were included | Not clear | Results reported as percentages although adjustment for age, sex and follow up period is reported separately. |
| 16b: Report category boundaries when continuous variables were categorized | No |  |
| 16c: If relevant, consider translating estimates of relative risk into absolute risk for a meaningful time period | NA | Study aimed for a comparison of quality of life and functional outcome; not applicable to translate to absolute risk. |
| 17: Other analyses  Report other analyses done—e.g. analyses of subgroups and interactions, and sensitivity analyses | Yes | Also examined outcomes for community and outpatient groups and compared results to normative data for relevant population. |
| Discussion | | |
| 18: Key results  Summarise key results with reference to study objectives | Yes | Section Discussion, p62. |
| 19: Limitations  Discuss limitations of the study, taking into account sources of potential bias or imprecision. Discuss both direction and magnitude of any potential bias | Yes | Acknowledged limitations of lack of preoperative data, high number of refusal to participate/loss to follow up, lack of controls in relation to co-morbidities and sample size. |
| 20: Interpretation  Give a cautious overall interpretation of results considering objectives, limitations, multiplicity of analyses, results from similar studies, and other relevant evidence | Yes | Section Discussion, p62 – 63. |
| 21: Generalisability  Discuss the generalisability (external validity) of the study results | Yes | Section Discussion, p62 0 63.  Particular caution urged in relation to co-morbidities. |
| Other information | | |
| 22: Funding  Give the source of funding and the role of the funders for the present study and, if applicable, for the original study on which the present article is based | Yes | “Partial funding for this research was received from the Australian Orthopaedic Association Research Foundation” (p63). |

| STUDY REFERENCE | |  |
| --- | --- | --- |
| ***Falavigna 2011*** | Enter Yes/No/Not clear or NA | Text that helped you make the decision, including page number |
| Title and abstract | | |
| 1a: Indicate the study’s design with a commonly used term in the title or the abstract | Not clear | Title states “a prospective study” but not what type. |
| 1b: Provide in the abstract an informative and balanced summary of what was done and what was found | Yes | Section Abstract (p399). |
| Introduction | | |
| 2: Background/rationale  Explain the scientific background and rationale for the investigation being reported | Yes | Opening paragraphs (p399 – 400). |
| 3: Objectives  State specific objectives, including any prespecified hypotheses | Yes | “to evaluate the impact of a deep wound infection after instrumented lumbar arthrodesis for symptomatic degenerative disc disease on functional outcome and patient satisfaction” (p400). |
| Methods | | |
| 4: Study design  Present key elements of study design early in the paper | Yes | Section Methods (p400 – 1). |
| 5: Setting  Describe the setting, locations, and relevant dates, including periods of recruitment, exposure, follow-up, and data collection | Not clear | No information on setting, locations or dates of study provided.  “median follow up duration was 22 months” (p401).  “The patients answered the entire questionnaire and the satisfaction question by themselves using a touchscreen computer questionnaire system without any interaction  with the medical or research staff” (p400). |
| Participants  6a: Give the eligibility criteria, and the sources and methods of case ascertainment and control selection. Give the rationale for the choice of cases and controls | Yes | “13 patients who developed a deep wound infection after lumbar spine decompression and instrumented arthrodesis for degenerative lumbar stenosis and instability. This group  was defined as the infection group. The control group was defined as patients without a postoperative infection” (p400). |
| 6b: For matched studies, give matching criteria and the number of controls per case | Yes | “The control group was matched to the infection group based on the surgical pathology, sex, and age at a ratio of 3:1” (p400). |
| 7: Variables  Clearly define all outcomes, exposures, predictors, potential confounders, and effect modifiers. Give diagnostic criteria, if applicable | Yes | “Validated instruments were used to evaluate pain, disability, quality of life, and anxiety and depression” (p400)  Instruments used were the numerical rating scale for pain, Oswestry Disability Index and SF-36 for HRQoL. |
| 8: Data sources/measurement  For each variable of interest, give sources of data and details of methods of assessment (measurement). Describe comparability of assessment methods if there is more than one group | Not clear | “The patients answered the entire questionnaire and the satisfaction question by themselves using a touchscreen computer questionnaire system without any interaction  with the medical or research staff” (p400)  Not clear if this was just at follow up or how pre-operative were gathered. |
| 9: Bias  Describe any efforts to address potential sources of bias | Not clear | Same surgeon and site but beyond this and matching criteria, no further information provided. |
| 10: Study size  Explain how the study size was arrived at | No | No information provided. |
| 11: Quantitative variables  Explain how quantitative variables were handled in the analyses. If applicable, describe which groupings were chosen and why | Not clear | “The comparative analyses between  the groups (infection and control) were conducted with the Fisher exact test for categorical variables and the Student  t-test or Mann-Whitney U-test for continuous variables, depending on the distribution” (p401)  No further information provided. |
| Statistical methods  12a: Describe all statistical methods, including those used to control for confounding | Yes | “The comparative analyses between  the groups (infection and control) were conducted with the Fisher exact test for categorical variables and the Student  t-test or Mann-Whitney U-test for continuous variables, depending on the distribution” (p401) |
| 12b: Describe any methods used to examine subgroups and interactions | NA | No subgroup analysis carried out. |
| 12c: Explain how missing data were addressed | NA | No missing data. |
| 12d: If applicable, explain how loss to follow up was addressed | NA | No loss to follow up reported. |
| 12d: If applicable, explain how matching of cases and controls was addressed | Yes | “The control group was matched to the infection group based on the surgical pathology, sex, and age at a ratio of 3:1” (p400). |
| 12e: Describe any sensitivity analyses | NA | No sensitivity analysis completed. |
| Results | | |
| Participants  13a: Report numbers of individuals at each stage of study— e.g. numbers potentially eligible, examined for eligibility, confirmed eligible, included in the study, completing follow-up, and analysed | No | Only final numbers provided (p401). |
| 13b: Give reasons for non-participation at each stage | No | Not provided. |
| 13c: Consider use of a flow diagram | No | Not provided. |
| Descriptive data  14a: Give characteristics of study participants (eg demographic, clinical, social) and information on exposures and potential confounders | Yes | Table 1 (p401). |
| 14b: Indicate number of participants with missing data for each variable of interest | NA | No missing data reported. |
| 15: Outcome data  Report numbers in each exposure category, or summary measures of exposure | Yes | Table 2 (p401). |
| Main results  16a: Give unadjusted estimates and, if applicable, confounder-adjusted estimates and their precision (eg, 95% confidence interval). Make clear which confounders were adjusted for and why they were included | Not clear | Figures in Table 2 maybe inferred to be unadjusted but not confirmed. |
| 16b: Report category boundaries when continuous variables were categorized | NA | Continuous variables not categorised. |
| 16c: If relevant, consider translating estimates of relative risk into absolute risk for a meaningful time period | NA | Not applicable due to outcome measure (HRQoL). |
| 17: Other analyses  Report other analyses done—e.g. analyses of subgroups and interactions, and sensitivity analyses | NA | No further analyses carried out. |
| Discussion | | |
| 18: Key results  Summarise key results with reference to study objectives | Yes | Section Discussion (p401 -2). |
| 19: Limitations  Discuss limitations of the study, taking into account sources of potential bias or imprecision. Discuss both direction and magnitude of any potential bias | No | No limitations acknowledged. |
| 20: Interpretation  Give a cautious overall interpretation of results considering objectives, limitations, multiplicity of analyses, results from similar studies, and other relevant evidence | Yes | Section Discussion (p401 -2). |
| 21: Generalisability  Discuss the generalisability (external validity) of the study results | Yes | Section Discussion (p401 -2). |
| Other information | | |
| 22: Funding  Give the source of funding and the role of the funders for the present study and, if applicable, for the original study on which the present article is based | Not clear | “Dr. Traynelis is consultant for and a patent holder in Medtronic” (p402). No further information provided. |

| STUDY REFERENCE | |  |
| --- | --- | --- |
| ***Guirro 2016*** | Enter Yes/No/Not clear or NA | Text that helped you make the decision, including page number |
| Title and abstract | | |
| 1a: Indicate the study’s design with a commonly used term in the title or the abstract | Not clear | “3000 prospective TKA cohort, 45 superficial infections were to a control group of 629 TKA without complication” (p3088)  No specific mention of study design. |
| 1b: Provide in the abstract an informative and balanced summary of what was done and what was found | Yes | Section Abstract (p3088). |
| Introduction | | |
| 2: Background/rationale  Explain the scientific background and rationale for the investigation being reported | Yes | Section Introduction (p3088-9). |
| 3: Objectives  State specific objectives, including any prespecified hypotheses | Yes | “…hypothesized that the superficial infection of a surgical wound following a successfully treated TKA does not lead to lower functional outcome and lower health related quality of life (HRQoL) in comparison with the TKA without other complications at long term follow up.” (p3089) |
| Methods | | |
| 4: Study design  Present key elements of study design early in the paper | Yes | Section Materials and Methods (p3089-90). |
| 5: Setting  Describe the setting, locations, and relevant dates, including periods of recruitment, exposure, follow-up, and data collection | Yes | “A prospective cohprt of 3000 TKAs performed from September 2005 to April 2010 in the same operating room by the same six member team of surgeons was constituted” (p3089)  “Patients were evaluated by their surgeon at 2 weeks, 2 months, 6 months, 12 months after surgery and yearly thereafter” (p3089)  “Health-related Quality of life (HRQoL) was assessed using the Short Form 36 (SF-36)” (p3090) |
| Participants  6a: Give the eligibility criteria, and the sources and methods of case ascertainment and control selection. Give the rationale for the choice of cases and controls | Yes | “All patients diagnosed as having a superficial infection were included”  “The control group was composed of consecutive TKA procedures carried out between 2007 and 2008 from the same cohort of TKAs to achieve a matched control group” (p3090) |
| 6b: For matched studies, give matching criteria and the number of controls per case | Not clear | Matching criteria given as “demographic data and co-morbidities” (p3090), but do not specify what these were. No information provided on number of controls per case. |
| 7: Variables  Clearly define all outcomes, exposures, predictors, potential confounders, and effect modifiers. Give diagnostic criteria, if applicable | Yes | Outcome measures included health related quality of life, cognitive status, knee function and fulfilment of expectations.  Superficial infection diagnosed according to CDC criteria. |
| 8: Data sources/measurement  For each variable of interest, give sources of data and details of methods of assessment (measurement). Describe comparability of assessment methods if there is more than one group | Yes | Health related quality of life: SF-36  Cognitive status: Short Portable Mental Status Questionnaire  Knee function: Knee Society score  Fulfilment of expectations: Hospital for Special Surgery Knee Replacement Expectations Survey |
| 9: Bias  Describe any efforts to address potential sources of bias | Not clear | Same surgical protocol followed for all patients but beyond this no further information provided. |
| 10: Study size  Explain how the study size was arrived at | Not clear | Inference is study recruited to date limit, not sample size. |
| 11: Quantitative variables  Explain how quantitative variables were handled in the analyses. If applicable, describe which groupings were chosen and why | Yes | Tabular data also provide counts of cases and controls. |
| Statistical methods  12a: Describe all statistical methods, including those used to control for confounding | Not clear | “Chi-square analysis was used to compare categorical variables, and the Man-Whitney U test was used to compare quantitative variables” (p3091)  No further information provided about controlling for confounders. |
| 12b: Describe any methods used to examine subgroups and interactions | Yes | As above  No subgroup analysis completed. |
| 12c: Explain how missing data were addressed | Not clear | No information provided. |
| 12d: If applicable, explain how loss to follow up was addressed | Not clear | No information provided. |
| 12d: If applicable, explain how matching of cases and controls was addressed | Yes | Matching criteria given as “demographic data and co-morbidities” (p3090), but do not specify what these were. |
| 12e: Describe any sensitivity analyses | NA | No sensitivity analysis completed. |
| Results | | |
| Participants  13a: Report numbers of individuals at each stage of study— e.g. numbers potentially eligible, examined for eligibility, confirmed eligible, included in the study, completing follow-up, and analysed | Yes | Narrative overview in Results (p3091-2). |
| 13b: Give reasons for non-participation at each stage | Yes | Narrative overview in Results (p3091-2). |
| 13c: Consider use of a flow diagram | No | Not provided. |
| Descriptive data  14a: Give characteristics of study participants (eg demographic, clinical, social) and information on exposures and potential confounders | Yes | Table 1, p3091. |
| 14b: Indicate number of participants with missing data for each variable of interest | Not clear | Although may be inferred from narrative in Results (p3901-2). |
| 15: Outcome data  Report numbers in each exposure category, or summary measures of exposure | Yes | Tables 3 and 4, p3092. |
| Main results  16a: Give unadjusted estimates and, if applicable, confounder-adjusted estimates and their precision (eg, 95% confidence interval). Make clear which confounders were adjusted for and why they were included | Not clear | Main outcome measures provided in Table 4 (p3092) although it is not clear if these are unadjusted. |
| 16b: Report category boundaries when continuous variables were categorized | Yes | In section Outcome measurements at final follow up, (p3090). |
| 16c: If relevant, consider translating estimates of relative risk into absolute risk for a meaningful time period | NA | Not relevant given outcome measures. |
| 17: Other analyses  Report other analyses done—e.g. analyses of subgroups and interactions, and sensitivity analyses | NA | No other analysis completed. |
| Discussion | | |
| 18: Key results  Summarise key results with reference to study objectives | Yes | Section Discussion (p3092-3). |
| 19: Limitations  Discuss limitations of the study, taking into account sources of potential bias or imprecision. Discuss both direction and magnitude of any potential bias | Yes | “sample size is small…some problems with wound healing may have been underestimated” (p3093). |
| 20: Interpretation  Give a cautious overall interpretation of results considering objectives, limitations, multiplicity of analyses, results from similar studies, and other relevant evidence | Yes | Section Discussion (p3092-3). |
| 21: Generalisability  Discuss the generalisability (external validity) of the study results | Not clear | States implications for practice but not in terms of external validity. |
| Other information | | |
| 22: Funding  Give the source of funding and the role of the funders for the present study and, if applicable, for the original study on which the present article is based | Yes | “This study was not funded by any company or other source” (p3094). |

| STUDY REFERENCE | |  |
| --- | --- | --- |
| ***Haddad 2018*** | Enter Yes/No/Not clear or NA | Text that helped you make the decision, including page number |
| Title and abstract | | |
| 1a: Indicate the study’s design with a commonly used term in the title or the abstract | Yes | “a matched control study” in title (p2518) |
| 1b: Provide in the abstract an informative and balanced summary of what was done and what was found | Yes | Section Abstract (p2518) |
| Introduction | | |
| 2: Background/rationale  Explain the scientific background and rationale for the investigation being reported | Yes | Section Introduction (p2519) |
| 3: Objectives  State specific objectives, including any prespecified hypotheses | Yes | “primary objective was to investigate the impact deep SSI has on ASD on Patient Reported Outcome Measures (PROMs) at different time intervals…secondary objectives were to analyse the associated morbidity of SSI and its impact on deformity correction” (p2519)  “Our hypothesis is that successfully treated deep SSI does not alter the functional outcome at 2 years even though it is likely to be associated with increased short term morbidity” (p2519). |
| Methods | | |
| 4: Study design  Present key elements of study design early in the paper | Yes | Section Methods (p2519) |
| 5: Setting  Describe the setting, locations, and relevant dates, including periods of recruitment, exposure, follow-up, and data collection | Yes | “six European centres from four different countries sharing a common ASD comprehensive database” (p2519)  “Between January 2010 and January 2016” (p2520)  “Patients were assessed at established time intervals (preoperatively, 6, 12, and 24 months post operatively)” (p2519). |
| Participants  6a: Give the eligibility criteria, and the sources and methods of case ascertainment and control selection. Give the rationale for the choice of cases and controls | Yes | “All adult patients who had undergone posterior instrumented spinal fusion for ASD with a minimum of 2 year follow up were included”  “Patients who had undergone treatment for a deep SSI formed the case group. They were accordingly matched to controls” (p2519). |
| 6b: For matched studies, give matching criteria and the number of controls per case | Yes | “gender, age, American Society of Anaesthesiologists Score (by categories 0-1, 2, 3-4), revision vs primary surgery, extent of fusion and the use of tri columnar osteotomies” (p2519)  “…1:3 matching proportion” (p2520). |
| 7: Variables  Clearly define all outcomes, exposures, predictors, potential confounders, and effect modifiers. Give diagnostic criteria, if applicable | Yes | Demographic and surgical variables  Heath related quality of life  Deep SSI diagnosed via CDC diagnostic criteria. |
| 8: Data sources/measurement  For each variable of interest, give sources of data and details of methods of assessment (measurement). Describe comparability of assessment methods if there is more than one group | Yes | ASD database  Numerical rating scale for back pain and leg pain  Oswestry Disability Index  36 Item Short Form Health Survey (SF-36)  Core Outcomes Measures Index  Scoliosis Research Society 22 Score (SRS-22 Score). |
| 9: Bias  Describe any efforts to address potential sources of bias | Yes | “The study contained a homogenous diagnostic and surgical population that was further matched using demographic and surgical variables known to affect both infection and outcome scores” (p2526). |
| 10: Study size  Explain how the study size was arrived at | Yes | “All adult patients who had undergone posterior instrumented spinal fusion for ASD with a minimum of 2 year follow up were included”(p2519) |
| 11: Quantitative variables  Explain how quantitative variables were handled in the analyses. If applicable, describe which groupings were chosen and why | Yes | Tabular data also provide counts of cases and controls. |
| Statistical methods  12a: Describe all statistical methods, including those used to control for confounding | Yes | “Descriptive and bivariate comparisons of demographic variables were performed between cases and controls using the independent t test for continuous variable, and Fischer’ exact test for the categorical variables” (p2520). |
| 12b: Describe any methods used to examine subgroups and interactions | Yes | In section Results, p2521. |
| 12c: Explain how missing data were addressed | NA | No missing data. |
| 12d: If applicable, explain how loss to follow up was addressed | Yes | One patient lost to follow up due to death. “We accordingly removed this patients and its paired controls from the radiological and clinical outcomes analysis” (p2521). |
| 12d: If applicable, explain how matching of cases and controls was addressed | Yes | “…the remaining 421 patients, 391 had not suffered from any postoperative infection and were available for matching. We could yield a 1:3 matching proportion after applying the six matching criteria. As such we had a 20:60 case-control cohort available” (p2520). |
| 12e: Describe any sensitivity analyses | NA | None completed. |
| Results | | |
| Participants  13a: Report numbers of individuals at each stage of study— e.g. numbers potentially eligible, examined for eligibility, confirmed eligible, included in the study, completing follow-up, and analysed | Yes | Section Results provides a narrative overview (p2520-1). |
| 13b: Give reasons for non-participation at each stage | Yes | Section Results provides a narrative overview (p2520-1). |
| 13c: Consider use of a flow diagram | Yes | Provided as Figure a, p2520. |
| Descriptive data  14a: Give characteristics of study participants (eg demographic, clinical, social) and information on exposures and potential confounders | Yes | Table 1, p2522. |
| 14b: Indicate number of participants with missing data for each variable of interest | NA | No missing data reported. |
| 15: Outcome data  Report numbers in each exposure category, or summary measures of exposure | Yes | Table 2, 3 and 4, p2522-3. |
| Main results  16a: Give unadjusted estimates and, if applicable, confounder-adjusted estimates and their precision (eg, 95% confidence interval). Make clear which confounders were adjusted for and why they were included | Not clear | Main outcome measures provided in Table 4 (p2524) although it is not clear if these are unadjusted. |
| 16b: Report category boundaries when continuous variables were categorized | NA | Continuous variables not categorised. |
| 16c: If relevant, consider translating estimates of relative risk into absolute risk for a meaningful time period | NA | Not relevant given outcome measures. |
| 17: Other analyses  Report other analyses done—e.g. analyses of subgroups and interactions, and sensitivity analyses | Yes | Demographic variables checked across each of the participating sites to explore site bias (p2521). |
| Discussion | | |
| 18: Key results  Summarise key results with reference to study objectives | Yes | Section Discussion(p2521). |
| 19: Limitations  Discuss limitations of the study, taking into account sources of potential bias or imprecision. Discuss both direction and magnitude of any potential bias | Yes | Non standard approach to treating SSI across several sites  Small sample size and lack of statistical power  Did not stratify infections in terms of virulence or course  Only included infections which were diagnosed early  Study could not account for impact of death (p2526-7). |
| 20: Interpretation  Give a cautious overall interpretation of results considering objectives, limitations, multiplicity of analyses, results from similar studies, and other relevant evidence | Yes | Section Discussion(p2521). |
| 21: Generalisability  Discuss the generalisability (external validity) of the study results | Yes | Section Discussion(p2521). |
| Other information | | |
| 22: Funding  Give the source of funding and the role of the funders for the present study and, if applicable, for the original study on which the present article is based | Yes | “The ESSG received research funds from DePuy-Synthes” (p2527). |

| STUDY REFERENCE | |  |
| --- | --- | --- |
| ***Kuhns 2015*** | Enter Yes/No/Not clear or NA | Text that helped you make the decision, including page number |
| Title and abstract | | |
| 1a: Indicate the study’s design with a commonly used term in the title or the abstract | Not clear | “…patients were individually matched with control patients” (p381)  No specific mention of study design. |
| 1b: Provide in the abstract an informative and balanced summary of what was done and what was found | Yes | Section Abstract, p381. |
| Introduction | | |
| 2: Background/rationale  Explain the scientific background and rationale for the investigation being reported | Yes | Paragraph 1 and 2 on p382. |
| 3: Objectives  State specific objectives, including any prespecified hypotheses | Yes | “We hypothesized that patients with wound infections following DCF would report decreased QOL and incur elevated health care costs” (p382). |
| Methods | | |
| 4: Study design  Present key elements of study design early in the paper | Yes | Section Methods (p382-3). |
| 5: Setting  Describe the setting, locations, and relevant dates, including periods of recruitment, exposure, follow-up, and data collection | Yes | “All patients who underwent dorsal cervical spine fusions from 2008 to 2012 were identified. Included were patients with a minimum of 5-month follow up who experienced a deep wound infection” (p382).  “Patient reported outcomes…Patient Health Questionnaire and Pain Disability Questionnaire…were established through the institutional Knowledge Program” (p382). |
| Participants  6a: Give the eligibility criteria, and the sources and methods of case ascertainment and control selection. Give the rationale for the choice of cases and controls | Yes | “All patients who underwent dorsal cervical spine fusions from 2008 to 2012 were identified. Included were patients with a minimum of 5-month follow up who experienced a deep wound infection” (p382). |
| 6b: For matched studies, give matching criteria and the number of controls per case | Yes | “…patients were individually matched to control patients of the same sex, age ± 5 years, body mass index (BMI) ± 5kg/m^2^, same operating surgeon, same instrumentation, date of surgery ± 2 years, and duration of follow up” (p382). |
| 7: Variables  Clearly define all outcomes, exposures, predictors, potential confounders, and effect modifiers. Give diagnostic criteria, if applicable | Yes | Patients reported outcomes  Cost data  “…we also controlled for cervical levels operated on; comorbidities including hypertension, coronary artery disease, hyperlipidemia, and diabetes; and preoperative medications” (p382).  “…deep wound infection as defined by the Centers for Disease Control and Prevention” (p382). |
| 8: Data sources/measurement  For each variable of interest, give sources of data and details of methods of assessment (measurement). Describe comparability of assessment methods if there is more than one group | Yes | Patient reported outcomes collected via EQ-5D, VAS, Patient Health Questionnaire and Pain Disability Questionnaire.  Cost data collected from electronic medical records. |
| 9: Bias  Describe any efforts to address potential sources of bias | Yes | “…we also controlled for cervical levels operated on; comorbidities including hypertension, coronary artery disease, hyperlipidemia, and diabetes; and preoperative medications” (p382). |
| 10: Study size  Explain how the study size was arrived at | Not clear | Recruitment by study dates (2008 – 2012) may be inferred. |
| 11: Quantitative variables  Explain how quantitative variables were handled in the analyses. If applicable, describe which groupings were chosen and why | Yes | Descriptive statistics as mean ± SD/ percentages, Paired T for parametric, Wilcoxon for nonparametric.  Analysed groups per and post op and also infected / non infected (p382-3). |
| Statistical methods  12a: Describe all statistical methods, including those used to control for confounding | Yes | Section Statistical Analysis (p382 – 3). |
| 12b: Describe any methods used to examine subgroups and interactions | Yes | “…infection and noninfection cohorts were compared with respect to numeric variables using independent sample t tests and categorical variables using Fisher exact test” (p383). |
| 12c: Explain how missing data were addressed | No | No information provided on missing data, although Table 2 shows some variables of interest were not collected from the full cohorts in the comparison. |
| 12d: If applicable, explain how loss to follow up was addressed | NA | Retrospective study design. |
| 12d: If applicable, explain how matching of cases and controls was addressed | Yes | “…patients were individually matched to control patients” (p382). |
| 12e: Describe any sensitivity analyses | NA | None completed. |
| Results | | |
| Participants  13a: Report numbers of individuals at each stage of study— e.g. numbers potentially eligible, examined for eligibility, confirmed eligible, included in the study, completing follow-up, and analysed | Yes | Section Demographics (p383). |
| 13b: Give reasons for non-participation at each stage | Yes | “29 patients were identified as having an infection, with 22 patients meeting the criteria of having a postoperative deep infection” (p383). |
| 13c: Consider use of a flow diagram | No | Not provided. |
| Descriptive data  14a: Give characteristics of study participants (eg demographic, clinical, social) and information on exposures and potential confounders | Yes | Table 1, p383. |
| 14b: Indicate number of participants with missing data for each variable of interest | Not clear | Although may be inferred from the numbers indicated from the “No.” columns in Table 2, p384. |
| 15: Outcome data  Report numbers in each exposure category, or summary measures of exposure | Yes | Table 1, p383. |
| Main results  16a: Give unadjusted estimates and, if applicable, confounder-adjusted estimates and their precision (eg, 95% confidence interval). Make clear which confounders were adjusted for and why they were included | Yes | Tables 2, 3 and 4 (p384).  Costs adjusted for direct/indirect costs and converted to 2013 dollars. |
| 16b: Report category boundaries when continuous variables were categorized | NA | Continuous variables not categorised. |
| 16c: If relevant, consider translating estimates of relative risk into absolute risk for a meaningful time period | NA | Not relevant for study outcomes. |
| 17: Other analyses  Report other analyses done—e.g. analyses of subgroups and interactions, and sensitivity analyses | Yes | Costs for different level of fusion (1-2 level fusion and 3 level fusion) (p383). |
| Discussion | | |
| 18: Key results  Summarise key results with reference to study objectives | Yes | Section Patient Reported Outcomes and Costs (p383). |
| 19: Limitations  Discuss limitations of the study, taking into account sources of potential bias or imprecision. Discuss both direction and magnitude of any potential bias | Yes | Retrospective nature of study  Short follow up (1 year)  Medicare reimbursement used for costs. |
| 20: Interpretation  Give a cautious overall interpretation of results considering objectives, limitations, multiplicity of analyses, results from similar studies, and other relevant evidence | Yes | Section Discussion (p383 – 5). |
| 21: Generalisability  Discuss the generalisability (external validity) of the study results | Yes | Section Discussion (p383 – 5). |
| Other information | | |
| 22: Funding  Give the source of funding and the role of the funders for the present study and, if applicable, for the original study on which the present article is based | Yes | “No grants or technical or corporate support were received in conducting this study or writing this manuscript” (p381). |

|  | |  |
| --- | --- | --- |
| ***Mok 2009*** | Enter Yes/No/Not clear or NA | Text that helped you make the decision, including page number |
| Title and abstract | | |
| 1a: Indicate the study’s design with a commonly used term in the title or the abstract | Yes | “A Matched Cohort Analysis” (p578) |
| 1b: Provide in the abstract an informative and balanced summary of what was done and what was found | Yes | Section Abstract, p578. |
| Introduction | | |
| 2: Background/rationale  Explain the scientific background and rationale for the investigation being reported | Yes | Background details provided on p578. |
| 3: Objectives  State specific objectives, including any prespecified hypotheses | Yes | “…to investigate clinical outcome in patients who underwent posterior spinal fusion complicated by deep wound infection in comparison to a matched cohort” (p578). |
| Methods | | |
| 4: Study design  Present key elements of study design early in the paper | Yes | Section Methods, p578 – 9. |
| 5: Setting  Describe the setting, locations, and relevant dates, including periods of recruitment, exposure, follow-up, and data collection | Yes | “retrospective study of 824 patients who underwent instrumented thoracolumbular spinal fusion using a posterior only or posterior and anterior (combined) approach in our institution from 1997 through 2002”  “…minimum 2-year clinical follow up and radiographic follow up at greater than 1 year after surgery” (p578 – 9). |
| Participants  6a: Give the eligibility criteria, and the sources and methods of case ascertainment and control selection. Give the rationale for the choice of cases and controls | Yes | As above and also:  “1:1 matched cohort (“control group”) consisting of patients who underwent fusion with posterior instrumentation within the same approximate period and did not develop deep wound infection was created, based on matching criteria including primary or revision status, length of fusion, diagnosis, and age” (p579). |
| 6b: For matched studies, give matching criteria and the number of controls per case | Yes | “…matching criteria including primary or revision status, length of fusion, diagnosis, and age” (p579).  16 cases and 16 controls (from Table 1, p579). |
| 7: Variables  Clearly define all outcomes, exposures, predictors, potential confounders, and effect modifiers. Give diagnostic criteria, if applicable | Yes | “Clinical outcome was measured by the physical domains of the SF-36…Secondary outcomes included radiographic fusion, need for reoperation, and characteristics of the infections” (p578)  “deep wound infection requiring treatment by debridement, positive intraoperative cultures from subfascial specimens” (p579). |
| 8: Data sources/measurement  For each variable of interest, give sources of data and details of methods of assessment (measurement). Describe comparability of assessment methods if there is more than one group | Yes | “…patient charts, computerized records, operative logs, and clinic notes” (p579)  “Medical Outcomes Study SF-36 general health survey version 2.0 at the most recent follow up visit or by mail” (p579). |
| 9: Bias  Describe any efforts to address potential sources of bias | Not clear | Beyond matching based on criteria above, no further information about specific sources of bias addressed. |
| 10: Study size  Explain how the study size was arrived at | Not clear | Although a date range may be implied from the narrative. |
| 11: Quantitative variables  Explain how quantitative variables were handled in the analyses. If applicable, describe which groupings were chosen and why | Yes | “Data were analysed using SPSS software…Dichotomous variables were compared using the Pearson X^2^ test. If the expected count for any cell was less than 5, Fisher exact test was used. Age, length of fusion, number of comorbidities, clinical outcomes scores, and length of follow up were analysed using Wilcoxon signed rank test for paired data” (p579). |
| Statistical methods  12a: Describe all statistical methods, including those used to control for confounding | Not clear | As above for statistical methods but no information provided on confounding. |
| 12b: Describe any methods used to examine subgroups and interactions | Yes | Outcomes also assessed according to infection (mono or polymicrobial). |
| 12c: Explain how missing data were addressed | NA | No missing data reported. |
| 12d: If applicable, explain how loss to follow up was addressed | NA | NA; retrospective study design. |
| 12d: If applicable, explain how matching of cases and controls was addressed | Yes | “1:1 matched cohort (“control group”) consisting of patients who underwent fusion with posterior instrumentation within the same approximate period and did not develop deep wound infection was created, based on matching criteria including primary or revision status, length of fusion, diagnosis, and age” (p579). |
| 12e: Describe any sensitivity analyses | NA | None completed. |
| Results | | |
| Participants  13a: Report numbers of individuals at each stage of study— e.g. numbers potentially eligible, examined for eligibility, confirmed eligible, included in the study, completing follow-up, and analysed | Yes | Section “Results” (p579 – 80). |
| 13b: Give reasons for non-participation at each stage | NA | No drop outs reported. |
| 13c: Consider use of a flow diagram | No | Not provided. |
| Descriptive data  14a: Give characteristics of study participants (eg demographic, clinical, social) and information on exposures and potential confounders | Yes | Table 1, p579. |
| 14b: Indicate number of participants with missing data for each variable of interest | NA | No missing data. |
| 15: Outcome data  Report numbers in each exposure category, or summary measures of exposure | Yes | Table 1 (p579) and Table 3 (p580). |
| Main results  16a: Give unadjusted estimates and, if applicable, confounder-adjusted estimates and their precision (eg, 95% confidence interval). Make clear which confounders were adjusted for and why they were included | Yes | Table 3 (p580), not applicable to adjustment. |
| 16b: Report category boundaries when continuous variables were categorized | NA | Continuous variables not categorized. |
| 16c: If relevant, consider translating estimates of relative risk into absolute risk for a meaningful time period | NA | Not relevant. |
| 17: Other analyses  Report other analyses done—e.g. analyses of subgroups and interactions, and sensitivity analyses | Not clear | Some information in narrative about early and late infection groups in relation to SF-36 scores although these are not presented in Table 3. |
| Discussion | | |
| 18: Key results  Summarise key results with reference to study objectives | Yes | Section Discussion, p581. |
| 19: Limitations  Discuss limitations of the study, taking into account sources of potential bias or imprecision. Discuss both direction and magnitude of any potential bias | Yes | Limitations acknowledged include:  - lack of preoperative data  - small sample sizes  - fusion determined by x-ray rather than gold standard CT. |
| 20: Interpretation  Give a cautious overall interpretation of results considering objectives, limitations, multiplicity of analyses, results from similar studies, and other relevant evidence | Yes | Section Discussion, p581. |
| 21: Generalisability  Discuss the generalisability (external validity) of the study results | Yes | Section Discussion, p581. |
| Other information | | |
| 22: Funding  Give the source of funding and the role of the funders for the present study and, if applicable, for the original study on which the present article is based | Yes | “No funds were received in support of this work” (p578). |

| STUDY REFERENCE | |  |
| --- | --- | --- |
| ***Pennington 2019*** | Enter Yes/No/Not clear or NA | Text that helped you make the decision, including page number |
| Title and abstract | | |
| 1a: Indicate the study’s design with a commonly used term in the title or the abstract | Not clear | “retrospective study”  “patients undergoing posterior lumbar decompression and fusion…were matched to controls” (p105)  Does not specifically state the matched cohort design although may be inferred from the narrative. |
| 1b: Provide in the abstract an informative and balanced summary of what was done and what was found | Yes | Section “Abstract”, p105. |
| Introduction | | |
| 2: Background/rationale  Explain the scientific background and rationale for the investigation being reported | Yes | Section “Introduction”, p105-6. |
| 3: Objectives  State specific objectives, including any prespecified hypotheses | Yes | “…to evaluate care costs and patient QOL…in a cohort of patients who underwent posterior lumbar decompression and fusion” (p106). |
| Methods | | |
| 4: Study design  Present key elements of study design early in the paper | Yes | Section “Methods” (p106). |
| 5: Setting  Describe the setting, locations, and relevant dates, including periods of recruitment, exposure, follow-up, and data collection | Yes | “all patients who underwent posterior lumbar decompression and fusion at our facility between 2008 and 2012. Patients were included if they had a minimum 6-month follow up time and experienced a deep wound infection” (p106).  “Pre-operative QOL data was acquired 30 days prior to the index operation. Postoperative data was collected at the 6-months follow up appointment” (p106). |
| Participants  6a: Give the eligibility criteria, and the sources and methods of case ascertainment and control selection. Give the rationale for the choice of cases and controls | Yes | “all patients who underwent posterior lumbar decompression and fusion at our facility” (p105)  “Patients were matched to gender, age ± 5 years, date of surgery, operating surgeon, and BMI ± 5. ..we also controlled for identified risks for SSI including hypertension, hyperlipidemia, and diabetes” (p106). |
| 6b: For matched studies, give matching criteria and the number of controls per case | Yes | “individually matched” (p106)  Criteria as above. |
| 7: Variables  Clearly define all outcomes, exposures, predictors, potential confounders, and effect modifiers. Give diagnostic criteria, if applicable | Yes | “The QOL outcome data used in this study included the following standardized PROs: Euro-Qol 5 dimensions (EQ-5D), Visual Analogue Scale (VAS), Patient Health Questionnaire 9 (PHQ – 9), and the Patient Disability Questionnaire (PDQ)” (p105). |
| 8: Data sources/measurement  For each variable of interest, give sources of data and details of methods of assessment (measurement). Describe comparability of assessment methods if there is more than one group | Yes | As above.  “Pre-operative QOL data was acquired 30 days prior to the index operation. Postoperative data was collected at the 6-months follow up appointment” (p106). |
| 9: Bias  Describe any efforts to address potential sources of bias | Not clear | Beyond matching based on criteria above, no further information about specific sources of bias addressed. |
| 10: Study size  Explain how the study size was arrived at | Not clear | Although a date range may be implied from the narrative. |
| 11: Quantitative variables  Explain how quantitative variables were handled in the analyses. If applicable, describe which groupings were chosen and why | Yes | “…we calculated QALY gain using the formula QALY = (Years of Life) x (Utility Value)” (p106)  “Descriptive statistics…were presented as means and standard deviations or counts with percent as appropriate. Pre and post operative changes in outcomes were analysed with paired t – tests and Wilcoxon Signed-Rank tests for parametric and non-parametric data…The infection and non-infection cohorts were compared with respect to numeric variables using independent sample t-tests and categorical variables using Fisher’s exact tests” (p106). |
| Statistical methods  12a: Describe all statistical methods, including those used to control for confounding | Not clear | As above for statistical methods but no information provided on confounding. |
| 12b: Describe any methods used to examine subgroups and interactions | NA | No subgroup analysis completed. |
| 12c: Explain how missing data were addressed | No | Missing data for some QoL in Table 2 but no narrative as to how this was addressed. |
| 12d: If applicable, explain how loss to follow up was addressed | No | No loss to follow up reported in narrative but data in Table 2 is not complete for n = 18 for all variables. |
| 12d: If applicable, explain how matching of cases and controls was addressed | Yes | “all patients who underwent posterior lumbar decompression and fusion at our facility” (p105)  “Patients were matched to gender, age ± 5 years, date of surgery, operating surgeon, and BMI ± 5. ..we also controlled for identified risks for SSI including hypertension, hyperlipidemia, and diabetes” (p106). |
| 12e: Describe any sensitivity analyses | NA | None completed. |
| Results | | |
| Participants  13a: Report numbers of individuals at each stage of study— e.g. numbers potentially eligible, examined for eligibility, confirmed eligible, included in the study, completing follow-up, and analysed | Yes | Section 3.1 “Patient sample” (p106-7). |
| 13b: Give reasons for non-participation at each stage | No | No loss to follow up reported in narrative but data in Table 2 is not complete for n = 18 for all variables. |
| 13c: Consider use of a flow diagram | No | Not provided. |
| Descriptive data  14a: Give characteristics of study participants (eg demographic, clinical, social) and information on exposures and potential confounders | Yes | Table 1, p107. |
| 14b: Indicate number of participants with missing data for each variable of interest | Yes | Table 2, p107. |
| 15: Outcome data  Report numbers in each exposure category, or summary measures of exposure | Yes | Table 1, p107. |
| Main results  16a: Give unadjusted estimates and, if applicable, confounder-adjusted estimates and their precision (eg, 95% confidence interval). Make clear which confounders were adjusted for and why they were included | Not clear | Table 2 details all estimates for QoL but no indication is given if these are adjusted or unadjusted. Narrative identifies controlling for identified SSI risks (p106) but this is not indicated in Table 2. |
| 16b: Report category boundaries when continuous variables were categorized | NA | Continuous variables not categorised. |
| 16c: If relevant, consider translating estimates of relative risk into absolute risk for a meaningful time period | NA | Not relevant. |
| 17: Other analyses  Report other analyses done—e.g. analyses of subgroups and interactions, and sensitivity analyses | NA | Analysis only completed on infected / non-infected groups. |
| Discussion | | |
| 18: Key results  Summarise key results with reference to study objectives | Yes | “We find that surgical site infections are associated with a 128% increase in total costs, but do not lead to a significant difference in QOL outcomes” (p108). |
| 19: Limitations  Discuss limitations of the study, taking into account sources of potential bias or imprecision. Discuss both direction and magnitude of any potential bias | Yes | Section 4.3 “Limitations” (p108 – 9). |
| 20: Interpretation  Give a cautious overall interpretation of results considering objectives, limitations, multiplicity of analyses, results from similar studies, and other relevant evidence | Yes | Section “Discussion” (p108). |
| 21: Generalisability  Discuss the generalisability (external validity) of the study results | Yes | Section “Discussion” (p108). |
| Other information | | |
| 22: Funding  Give the source of funding and the role of the funders for the present study and, if applicable, for the original study on which the present article is based | Yes | “Disclosure of funding: None” (p109). |

| STUDY REFERENCE | |  |
| --- | --- | --- |
| ***Perencevich 2003*** | Enter Yes/No/Not clear or NA | Text that helped you make the decision, including page number |
| Title and abstract | | |
| 1a: Indicate the study’s design with a commonly used term in the title or the abstract | No | “matched cohort design” first mentioned in Methods section (p196). |
| 1b: Provide in the abstract an informative and balanced summary of what was done and what was found | Yes | Opening paragraph (p196). |
| Introduction | | |
| 2: Background/rationale  Explain the scientific background and rationale for the investigation being reported | Yes | Paragraphs 2 – 4, p196. |
| 3: Objectives  State specific objectives, including any prespecified hypotheses | Not clear | “to compare the costs and illness of patients with an SSI to matched patients who had surgery during the same period but in whom an SSI did not develop” (p196)  No further objectives or hypotheses stated. |
| Methods | | |
| 4: Study design  Present key elements of study design early in the paper | Yes | Section “Methods” (p196-7). |
| 5: Setting  Describe the setting, locations, and relevant dates, including periods of recruitment, exposure, follow-up, and data collection | Yes | “drawn from adult members of the Harvard Vanguard Medical Associates…who had undergone a nonobstetric inpatient or outpatient operating room procedure at Brigham and Women’s Hospital from May 18, 1997, through October 31, 1998” (p196)  Data collection used “automated medical record screening”, pharmacy records and post discharge surveillance data. |
| Participants  6a: Give the eligibility criteria, and the sources and methods of case ascertainment and control selection. Give the rationale for the choice of cases and controls | Yes | “adult…who had undergone a nonobstetric inpatient or outpatient operating room procedure at Brigham and Women’s Hospital from May 18, 1997, through October 31, 1998” (p196)  “enrolled 5 – 7 weeks after surgery” (p197)  “Case-patients were individually matched on surgery type, age and duration of surgical procedure in a ratio of one case-patient to two other members of the cohort” (p197). |
| 6b: For matched studies, give matching criteria and the number of controls per case | Yes | “Case-patients were individually matched on surgery type, age and duration of surgical procedure in a ratio of one case-patient to two other members of the cohort” (p197). |
| 7: Variables  Clearly define all outcomes, exposures, predictors, potential confounders, and effect modifiers. Give diagnostic criteria, if applicable | Yes | Outcomes measured using National Health Interview Survey questions, QoL measured using SF-12. NNIS 30-day surveillance criteria used for SSI diagnosis. |
| 8: Data sources/measurement  For each variable of interest, give sources of data and details of methods of assessment (measurement). Describe comparability of assessment methods if there is more than one group | Yes | “Participants were enrolled 5 – 7 weeks after surgery. All case-patients and matched pairs were mailed a 49-item questionnaire” (p197)  4 administrative databases also used for data collection. |
| 9: Bias  Describe any efforts to address potential sources of bias | Not clear | Beyond matching based on criteria above, no further information about specific sources of bias addressed. |
| 10: Study size  Explain how the study size was arrived at | Yes | “During the anticipated study period, 3,00 surgeries would be estimated to be performed and, given a 2.8% risk for infection…, 84 SSI would be recognised after discharge. This gave a power of 0.89 to detect ≥ 5 days lost from usual activities” (p198). |
| 11: Quantitative variables  Explain how quantitative variables were handled in the analyses. If applicable, describe which groupings were chosen and why | Yes | “Student t test, Wilcoxon rank-sum test, or Fisher exact test were used, where appropriate, for univariate comparisons…Cases and matched control were compared using the Wilcoxon signed-rank test for continuous outcomes with non-normal distributions, continuous linear regression by forcing the matching variable into the model for normally distributed variables, or the Cochran-Mantel-Haenszel for matched binary variables” (p197 – 8). |
| Statistical methods  12a: Describe all statistical methods, including those used to control for confounding | Yes | Section Statistics (p197 – 8). |
| 12b: Describe any methods used to examine subgroups and interactions | NA | No subgroup analysis completed beyond case and control comparisons. |
| 12c: Explain how missing data were addressed | Not clear | “(65%) of 267 questionnaires were returned” (p199). No further narrative to explain how these were handled in the analyses. |
| 12d: If applicable, explain how loss to follow up was addressed | Not clear | “(65%) of 267 questionnaires were returned” (p199). No further narrative to explain how these were handled in the analyses. |
| 12d: If applicable, explain how matching of cases and controls was addressed | Yes | “Case-patients were individually matched on surgery type, age and duration of surgical procedure in a ratio of one case-patient to two other members of the cohort” (p197). |
| 12e: Describe any sensitivity analyses | NA | None completed. |
| Results | | |
| Participants  13a: Report numbers of individuals at each stage of study— e.g. numbers potentially eligible, examined for eligibility, confirmed eligible, included in the study, completing follow-up, and analysed | Yes | Section Results (p198 – 200). |
| 13b: Give reasons for non-participation at each stage | Not clear | Numbers of non participation at each stage provided but no reasons for these are provided. |
| 13c: Consider use of a flow diagram | No | Not provided. |
| Descriptive data  14a: Give characteristics of study participants (eg demographic, clinical, social) and information on exposures and potential confounders | Yes | Table 2, p199. |
| 14b: Indicate number of participants with missing data for each variable of interest | Yes | Table 3, p200. |
| 15: Outcome data  Report numbers in each exposure category, or summary measures of exposure | Yes | Table 3, p200. |
| Main results  16a: Give unadjusted estimates and, if applicable, confounder-adjusted estimates and their precision (eg, 95% confidence interval). Make clear which confounders were adjusted for and why they were included | Not clear | Table 3 outlines main QoL results (of interest for this Review), but does not provide information regarding whether these were adjusted or unadjusted. |
| 16b: Report category boundaries when continuous variables were categorized | NA | Continuous variables not categorized. |
| 16c: If relevant, consider translating estimates of relative risk into absolute risk for a meaningful time period | Yes | Converted cost data to calculate excess costs associated with SSI, extra days in bed also calculated for SSI. |
| 17: Other analyses  Report other analyses done—e.g. analyses of subgroups and interactions, and sensitivity analyses | NA | No subgroup analyses completed. |
| Discussion | | |
| 18: Key results  Summarise key results with reference to study objectives | Yes | Section Discussion (p200 – 201). |
| 19: Limitations  Discuss limitations of the study, taking into account sources of potential bias or imprecision. Discuss both direction and magnitude of any potential bias | Yes | Limitations acknowledged include inability to assess all societal costs of SSI and selection bias associated with stringent matching. |
| 20: Interpretation  Give a cautious overall interpretation of results considering objectives, limitations, multiplicity of analyses, results from similar studies, and other relevant evidence | Yes | Section Discussion (p200 – 201). |
| 21: Generalisability  Discuss the generalisability (external validity) of the study results | Yes | Section Discussion (p200 – 201). |
| Other information | | |
| 22: Funding  Give the source of funding and the role of the funders for the present study and, if applicable, for the original study on which the present article is based | Yes | “Funded by a grant from the Harvard Pilgrim Health Care Foundation and the Centers for Disease Control and Prevention Eastern Massachusetts Prevention Epicenter cooperative agreement UR8/CCU115079” (p202). |

| STUDY REFERENCE | |  |
| --- | --- | --- |
| ***Petilon 2012*** | Enter Yes/No/Not clear or NA | Text that helped you make the decision, including page number |
| Title and abstract | | |
| 1a: Indicate the study’s design with a commonly used term in the title or the abstract | Yes | Title “A Case-Control Study” (p1370). |
| 1b: Provide in the abstract an informative and balanced summary of what was done and what was found | Yes | Section Abstract (p1370). |
| Introduction | | |
| 2: Background/rationale  Explain the scientific background and rationale for the investigation being reported | Yes | Paragraphs 1 – 2 (p1370). |
| 3: Objectives  State specific objectives, including any prespecified hypotheses | Yes | “The purpose of this study was to evaluate the 2-year health-related quality of life (HRQOL) measures of patients who have undergone instrumented spinal fusion complicated by deep wound infection, necessitating irrigation and debridement” (p1370). |
| Methods | | |
| 4: Study design  Present key elements of study design early in the paper | Yes | Section Materials and Methods (p1371). |
| 5: Setting  Describe the setting, locations, and relevant dates, including periods of recruitment, exposure, follow-up, and data collection | Yes | “2001 to 2008” (p1371)  “patients who underwent lumbar spinal fusion” (p1371)  “patients who underwent lumbar spinal fusion with complete preoperative and 2-year postoperative outcome measures and had acute (≤3 mo) postoperative deep wound infections…necessitating irrigation and debridement” (p1371). |
| Participants  6a: Give the eligibility criteria, and the sources and methods of case ascertainment and control selection. Give the rationale for the choice of cases and controls | Yes | “patients who underwent lumbar spinal fusion with complete preoperative and 2-year postoperative outcome measures and had acute (≤3 mo) postoperative deep wound infections…necessitating irrigation and debridement” (p1371)  “Patients who had surgery for trauma, tumor, or osteomyelitis were excluded” (p1731)  “Propensity scoring technique was then used to match the patients with surgical wound infection with a control group who did not have postoperative wound infections” (p1371). |
| 6b: For matched studies, give matching criteria and the number of controls per case | Yes | “Sex, age, body mass index, smoking status, indication for fusion, type of fusion, and number of levels fused as well as preoperative ODI, 36-Item Short Form Health Survey PCS, 36-Item Short Form Health Survey MCS, and back and leg pain were used to generate the control group (p1371). |
| 7: Variables  Clearly define all outcomes, exposures, predictors, potential confounders, and effect modifiers. Give diagnostic criteria, if applicable | Yes | “HRQOL measures collected and reviewed in this study included the Oswestry Disability Index (ODI), the Medical Outcomes Study Short Form 36…and numeric rating scales for back and leg pain” (p1371).  SSI diagnosed by CDC diagnostic criteria for deep wound infections. |
| 8: Data sources/measurement  For each variable of interest, give sources of data and details of methods of assessment (measurement). Describe comparability of assessment methods if there is more than one group | Yes | As above |
| 9: Bias  Describe any efforts to address potential sources of bias | Not clear | Beyond matching based on criteria above, no further information about specific sources of bias addressed. |
| 10: Study size  Explain how the study size was arrived at | Yes | “met inclusion criteria” during the date range for data collection (p1371). |
| 11: Quantitative variables  Explain how quantitative variables were handled in the analyses. If applicable, describe which groupings were chosen and why | Yes | “Student t test…significant difference between continuous demographic variables…HRQOL measure scores within and between study groups” (p1371)  “Fisher exact test for categorical variables…Pearson correlations..to determine any correlations between 2-year HRQOL measures and number of debridements, days between infection diagnosis and debridement, the use antibiotic beads, and the need for flap closure” (p1371). |
| Statistical methods  12a: Describe all statistical methods, including those used to control for confounding | Yes | As above. |
| 12b: Describe any methods used to examine subgroups and interactions | Yes | “Pearson correlations...to determine any correlations between 2-year HRQOL measures and number of debridements, days between infection diagnosis and debridement, the use antibiotic beads, and the need for flap closure” (p1371). |
| 12c: Explain how missing data were addressed | NA | No missing data. |
| 12d: If applicable, explain how loss to follow up was addressed | NA | No loss to follow up. |
| 12d: If applicable, explain how matching of cases and controls was addressed | Yes | “Propensity scoring technique” (p1371). |
| 12e: Describe any sensitivity analyses | NA | No sensitivity analyses completed. |
| Results | | |
| Participants  13a: Report numbers of individuals at each stage of study— e.g. numbers potentially eligible, examined for eligibility, confirmed eligible, included in the study, completing follow-up, and analysed | Yes | Paragraph 1 in Section Results (p1371). |
| 13b: Give reasons for non-participation at each stage | NA | All patient data complete. |
| 13c: Consider use of a flow diagram | No | Not provided. |
| Descriptive data  14a: Give characteristics of study participants (eg demographic, clinical, social) and information on exposures and potential confounders | Yes | Table 1, p1372. |
| 14b: Indicate number of participants with missing data for each variable of interest | NA | Data complete for all 30 cases and controls. |
| 15: Outcome data  Report numbers in each exposure category, or summary measures of exposure | Yes | Tables 1 and 2 (p1371). |
| Main results  16a: Give unadjusted estimates and, if applicable, confounder-adjusted estimates and their precision (eg, 95% confidence interval). Make clear which confounders were adjusted for and why they were included | Not clear | Unclear from data presented if confounders were controlled for. |
| 16b: Report category boundaries when continuous variables were categorized | Yes | “minimum clinically important difference (MCID)…threshold for ODI is 12.8, 4.9 for the 36-Item Short Form Health Survey PCS, and 1.2 for back pain and 1.6 for leg pain” (p1371). |
| 16c: If relevant, consider translating estimates of relative risk into absolute risk for a meaningful time period | NA | Not applicable given outcome measures (HRQoL). |
| 17: Other analyses  Report other analyses done—e.g. analyses of subgroups and interactions, and sensitivity analyses | Yes | “Pearson’s correlation analysis…between number of debridements…days between infection diagnosis and debridement…use of beads…need for flap closure “(p1371-2). |
| Discussion | | |
| 18: Key results  Summarise key results with reference to study objectives | Yes | Paragraph three in section Discussion (p1373). |
| 19: Limitations  Discuss limitations of the study, taking into account sources of potential bias or imprecision. Discuss both direction and magnitude of any potential bias | Yes | Limitations acknowledged include:  - retrospective design  - small sample size  - no routine CT scan of fusions therefore undetected non-union may have influenced study results. |
| 20: Interpretation  Give a cautious overall interpretation of results considering objectives, limitations, multiplicity of analyses, results from similar studies, and other relevant evidence | Yes | Section Discussion (p1373). |
| 21: Generalisability  Discuss the generalisability (external validity) of the study results | Yes | Section Discussion (p1373). |
| Other information | | |
| 22: Funding  Give the source of funding and the role of the funders for the present study and, if applicable, for the original study on which the present article is based | Not clear | “Corporate/Industry funds were received to support this work” (p1370) but no further information about which author or the role of the funder in the research. |

| STUDY REFERENCE | |  |
| --- | --- | --- |
| ***Whitehouse 2019*** | Enter Yes/No/Not clear or NA | Text that helped you make the decision, including page number |
| Title and abstract | | |
| 1a: Indicate the study’s design with a commonly used term in the title or the abstract | Yes | “A pairwise-matched (1:1) case-control study within a cohort” (p183). |
| 1b: Provide in the abstract an informative and balanced summary of what was done and what was found | Yes | Section Abstract (p183). |
| Introduction | | |
| 2: Background/rationale  Explain the scientific background and rationale for the investigation being reported | Yes | Paragraphs 1- 3, p183. |
| 3: Objectives  State specific objectives, including any prespecified hypotheses | Yes | “We conducted a study from an institutional perspective of patients from both a tertiary-care referral center and a community hospital to measure the impact of orthopaedic SSI on (1) length of stay; (2) incidence of readmission; (3) costs attributable to SSI; and (4) quality of life” (p183). |
| Methods | | |
| 4: Study design  Present key elements of study design early in the paper | Yes | Section Methods (p183-4). |
| 5: Setting  Describe the setting, locations, and relevant dates, including periods of recruitment, exposure, follow-up, and data collection | Yes | “patients from Duke University Medical Center…and Durham Regional Hospital” (p183)  “between January 1 and December 31, 1997. Case patients identified at DRH had their original operative procedure between January 1,1997 and June 1, 1998” (p184)  “prospective inpatient surveillance…the operating room registries of both hospitals were searched for the years 1997 to 1998” (p184)  “The quality of life questionnaire was administered by telephone interview” (p184). |
| Participants  6a: Give the eligibility criteria, and the sources and methods of case ascertainment and control selection. Give the rationale for the choice of cases and controls | Yes | “SSI were defined using Centers for Disease Control and Prevention (CDC) National Nosocomial Infection Surveillance (NNIS) criteria…A case patient was defined as any patient who has an SSI following an inpatient surgical procedure” (p184)  “For each case patient, a matched control was selected from a database containing information on all inpatients who had undergone orthopaedic surgery during the study period” (p184). |
| 6b: For matched studies, give matching criteria and the number of controls per case | Yes | As above and also “Variables used for matching included type of operative procedure, NNIS risk index, age within 5 years, date of surgery within the same year, and same surgeon” (p184). |
| 7: Variables  Clearly define all outcomes, exposures, predictors, potential confounders, and effect modifiers. Give diagnostic criteria, if applicable | Yes | Patient characteristics collected included date of broth, gender, race, dates of any hospitalizations during the follow up period, residence in a nursing home or rehabilitation center, and use of home healthcare services” (p184)  Hospitalisation data and accounting costs.  HRQoL collected via SF-36.  SSI diagnosed via CDC diagnostic criteria. |
| 8: Data sources/measurement  For each variable of interest, give sources of data and details of methods of assessment (measurement). Describe comparability of assessment methods if there is more than one group | Yes | “by review of each patient’s medical record” (p184)  “The quality of life questionnaire was administered by telephone interview” (p184)  “total direct costs were obtained from the hospital business office” (p184). |
| 9: Bias  Describe any efforts to address potential sources of bias | Not clear | Beyond matching based on criteria above, no further information about specific sources of bias addressed. |
| 10: Study size  Explain how the study size was arrived at | Not clear | Although recruitment by date may be inferred from the narrative. |
| 11: Quantitative variables  Explain how quantitative variables were handled in the analyses. If applicable, describe which groupings were chosen and why | Yes | Analysis using chi-square test for “Differences and similarities in characteristics of cases and controls “(p185)  “Point estimates and interquartile ranges for median differences in length of hospitalisation…Wilcoxon signed rank test” (p185)  “Measures of quality of life that were multidimensional were analysed using a multivariate analysis of variance” (p185). |
| Statistical methods  12a: Describe all statistical methods, including those used to control for confounding | Yes | As above. |
| 12b: Describe any methods used to examine subgroups and interactions | NA | No subgroup analysis completed beyond cases and controls comparison. |
| 12c: Explain how missing data were addressed | Not clear | Although removal from main data set may be inferred from the narrative. |
| 12d: If applicable, explain how loss to follow up was addressed | Not clear | Although removal from main data set may be inferred from the narrative. |
| 12d: If applicable, explain how matching of cases and controls was addressed | Yes | As 6a and also “best possible match using the preceding criteria was performed in a stepwise fashion. If more than one equivalent potential control existed, a single individual was randomly chosen using a random numbers chart. If no control patient met the above matching criteria, the corresponding case was rejected from the analysis” (p184). |
| 12e: Describe any sensitivity analyses | NA | No sensitivity analysis completed. |
| Results | | |
| Participants  13a: Report numbers of individuals at each stage of study— e.g. numbers potentially eligible, examined for eligibility, confirmed eligible, included in the study, completing follow-up, and analysed | Yes | Section Results (p185). |
| 13b: Give reasons for non-participation at each stage | Yes | Figure p185. |
| 13c: Consider use of a flow diagram | Yes | Figure p185. |
| Descriptive data  14a: Give characteristics of study participants (eg demographic, clinical, social) and information on exposures and potential confounders | Not clear | Only operative procedure characteristics provided on Table 1 (p185). No further information provided on demographic profiles of the cohorts. |
| 14b: Indicate number of participants with missing data for each variable of interest | NA | 23 cases and controls with complete data for HRQoL (Table 4, p186). |
| 15: Outcome data  Report numbers in each exposure category, or summary measures of exposure | Yes | Table 4, p186. |
| Main results  16a: Give unadjusted estimates and, if applicable, confounder-adjusted estimates and their precision (eg, 95% confidence interval). Make clear which confounders were adjusted for and why they were included | Not clear | No information provided on any adjustments undertaken. |
| 16b: Report category boundaries when continuous variables were categorized | NA | Continuous variables not categorised. |
| 16c: If relevant, consider translating estimates of relative risk into absolute risk for a meaningful time period | NA | Not applicable given outcome measures (HRQoL). |
| 17: Other analyses  Report other analyses done—e.g. analyses of subgroups and interactions, and sensitivity analyses | Not clear | Reports unmatched analysis of HRQoL scores but these are not reported. |
| Discussion | | |
| 18: Key results  Summarise key results with reference to study objectives | Yes | Section Discussion (p186 – 7). |
| 19: Limitations  Discuss limitations of the study, taking into account sources of potential bias or imprecision. Discuss both direction and magnitude of any potential bias | Yes | Limitations acknowledged include:  - matching didn’t always include same operative category  - inclusion of uncommon procedures  - number of patients lost to follow up  - diagnosis of SSI out with two study sites  - underestimate the impact of SSI due to 1 year follow up period. |
| 20: Interpretation  Give a cautious overall interpretation of results considering objectives, limitations, multiplicity of analyses, results from similar studies, and other relevant evidence | Yes | Section Discussion (p186 – 7). |
| 21: Generalisability  Discuss the generalisability (external validity) of the study results | Yes | Section Discussion (p186 – 7). |
| Other information | | |
| 22: Funding  Give the source of funding and the role of the funders for the present study and, if applicable, for the original study on which the present article is based | No | No information about funding provided. |

**TABLE S3:** Quality assesment of included RCTs

| STUDY REFERENCE | |  |
| --- | --- | --- |
| ***Parker 2018***  ***Details also taken from trial protocol Achten 2015*** (16) ***and RCT write up from Costa 2018*** (17) | Enter **Y**es/**P**robably **Y**es/**P**robably **N**o/**N**o/**N**o **I**nformation | Text that helped you make the decision, including page number |
| Domain 1: Risk of bias arising from the randomisation process | | |
| 1.1 Was the allocation sequence random? | Yes | “A randomisation sequence, stratified by trial centre and Gustilo and Anderson grade, will be produced and administered by a secure web-based service. The random allocation will be to either standard wound management or negative pressure wound therapy.” (p3, Achten 2015). |
| 1.2 Was the allocation sequence concealed until participants were enrolled and assigned to interventions? | Yes | “The allocation sequence will be generated by an independent randomisation centre—York CTU. Randomisation will be on a 1:1 basis, stratified by trial centre and Gustilo and Anderson grade—2, 3, or 3 with vascular injury requiring surgical repair…. participants will be  assigned to their treatment allocation at the end of the initial surgery, but before the wound dressing is applied. All modern operating theatres include a computer with  web-access; so a secure, 24 h, web-based randomisation system will be used to generate the treatment allocation intraoperatively” (p6, Achten 2015). |
| 1.3 Did baseline differences between intervention groups suggest a problem with the randomization process? | No | Only significant differences noted in demographics between Deep SSI and no deep SSI group were diabetes (11% vs 5%) and smokers (46% vs 31%) (Table 1, p1508). |
| *Risk-of-bias judgement for Domain 1* | | ***Low*** |
| Domain 2: Risk of bias due to deviations from the intended interventions | | |
| 2.1. Were participants aware of their assigned intervention during the trial? | Probably no | “Some patients may be unconscious, all will be distracted by the injury to their leg and its subsequent treatment, and all will have had large doses of opiates for pain relief, affecting their ability to process information. The majority of patients will, therefore, lack capacity to make a decision about participation in a research project at this stage.” (p5, Achten 2015). |
| 2.2. Were carers and people delivering the interventions aware of participants' assigned intervention during the trial? | Yes | Intervention was “wound dressings are clearly visible, the patients cannot be blinded to their treatment. In addition, the treating surgeons will also not be blind to the treatment,  but will take no part in the postoperative assessment of the patients” (p7, Achten 2015). |
| 2.3. If Y/PY/NI to 2.1 or 2.2: Were there deviations from the intended intervention that arose because of the trial context? | No information | Not enough information provided. |
| 2.4 If Y/PY to 2.3: Were these deviations likely to have affected the outcome? | N/A | 2.3 was NI |
| 2.5. If Y/PY/NI to 2.4: Were these deviations from intended intervention balanced between groups? | N/A | 2.4 was N/A |
| 2.6 Was an appropriate analysis used to estimate the effect of assignment to intervention? | Yes | “230 patients who consent to each group will provide 90% power to detect a difference  of eight points in DRI at 12 months at the 5% level” (p4, Achten 2015). |
| 2.7 If N/PN/NI to 2.6: Was there potential for a substantial impact (on the result) of the failure to analyse participants in the group to which they were randomized? | N/A | 2.6 was Yes |
| *Risk-of-bias judgement for Domain 2* | | ***Some concerns*** |
| Domain 3: Risk of bias due to missing outcome data | | |
| 3.1 Were data for this outcome available for all, or nearly all, participants randomized? | No | 311 randomised to NPWT; 220 analysed  314 standard treatment; 230 analysed (Figure 1, p2283, Costa 2018). |
| 3.2 If N/PN/NI to 3.1: Is there evidence that the result was not biased by missing outcome data? | Yes | “The secondary per-protocol (per-treatment) analysis of the DRI did not significantly differ from the primary intention-to treat  analysis; the difference between groups being −4.0(95% CI, −9.1 to 1.0) in favor of the standard dressings (P=.12)” (p2283, Costa 2018). |
| 3.3 If N/PN to 3.2: Could missingness in the outcome depend on its true value? | N/A | 3.2 was Yes |
| 3.4 If Y/PY/NI to 3.3: Is it likely that missingness in the outcome depended on its true value? | N/A | 3.3 was N/A |
| *Risk-of-bias judgement for Domain 3* | | ***Low*** |
| Domain 4: Risk of bias in measurement of the outcome | | |
| 4.1 Was the method of measuring the outcome inappropriate? | No | Quality of life (outcome of interest for this Review) was EQ-5D-3L and SF-12 |
| 4.2 Could measurement or ascertainment of the outcome have differed between intervention groups? | No | “Patient-reported outcomes (DRI, EQ-5D-  3L, SF-12) and self-reported complications were collected by questionnaire” (p2282, Costa 2018). |
| 4.3 If N/PN/NI to 4.1 and 4.2: Were outcome assessors aware of the intervention received by study participants? | No | “The functional outcome data will be collected  and entered onto the trial central database via  postal questionnaire by a research assistant/data clerk in the trial central office… reviewed independently by two experienced  assessors who are blind to the treatment allocation” (p7, Achten 2015). |
| 4.4 If Y/PY/NI to 4.3: Could assessment of the outcome have been influenced by knowledge of intervention received? | N/A | 4.3 was No |
| 4.5 If Y/PY/NI to 4.4: Is it likely that assessment of the outcome was influenced by knowledge of intervention received? | N/A | 4.4 was N/A |
| *Risk-of-bias judgement for Domain 4* | | ***Low*** |
| Domain 5: Risk of bias in selection of the reported result | | |
| 5.1 Were the data that produced this result analysed in accordance with a pre-specified analysis plan that was finalized before unblinded outcome data were available for analysis? | Yes | Reported analysis of RCT in Costa 2018 aligns with analysis plan of protocol from Achten 2015. |
| Is the numerical result being assessed likely to have been selected, on the basis of the results, from... |  |  |
| 5.2. ... multiple eligible outcome measurements (e.g. scales, definitions, time points) within the outcome domain? | No | QALY calculated per convention using “Dolan United Kingdom algorithm” (p1507, Parker 2018). |
| 5.3 ... multiple eligible analyses of the data? | No | As above. |
| *Risk-of-bias judgement for Domain 5* | | ***Low*** |
| **Overall risk-of-bias judgement** | | **Some concerns** |

| STROBE criteria | |  |
| --- | --- | --- |
| ***Parker 2018***  ***Details also taken from trial protocol Achten 2015*** (16) ***and RCT write up from Costa 2018*** (17) | Enter Yes/No/Not clear or NA | Text that helped you make the decision, including page number |
| Title and abstract | | |
| 1a: Indicate the study’s design with a commonly used term in the title or the abstract | No | Refers to a “study” and “economic outcomes” only in title and abstract. |
| 1b: Provide in the abstract an informative and balanced summary of what was done and what was found | Yes | Abstract, p1506 detailing aims, patients and methods, results and conclusions drawn. |
| Introduction | | |
| 2: Background/rationale  Explain the scientific background and rationale for the investigation being reported | Yes | Paragraphs 1 – 4 (p1506 – 7). |
| 3: Objectives  State specific objectives, including any prespecified hypotheses | Yes | “to explore the health economic implications of deep infection after open fracture” (p1507). |
| Methods | | |
| 4: Study design  Present key elements of study design early in the paper | Yes | Section Patient and Methods (p1507) with references to the original trial protocol from Achten et al (2015). |
| 5: Setting  Describe the setting, locations, and relevant dates, including periods of recruitment, exposure, follow-up, and data collection | Yes | “etreated in a United Kingdom Major Trauma Centre or Trauma Unit with joint orthopaedic and plastic surgical care” (p1507)  “from July 2012 through December 2015” (p2280, Costa 2018)  “Final outcome data were collected through November 2016” (p2280, Costa 2018). |
| Participants  6a: Give the eligibility criteria, and the sources and methods of case ascertainment and control selection. Give the rationale for the choice of cases and controls | Yes | “Eligible patients were aged 16 years or older and had a severe open fracture of the lower  limb... the operating surgeon assessed the wound and if it was decided that the wound could not be closed, the patient was considered  eligible for study entry” (2281, Costa 2018) |
| 6b: For matched studies, give matching criteria and the number of controls per case | Yes | “Two groups of patients were compared: those without, and those with, a diagnosis of deep surgical site infection (SSI), according to the Centres for Disease Control (CDC) definition” (p1507) |
| 7: Variables  Clearly define all outcomes, exposures, predictors, potential confounders, and effect modifiers. Give diagnostic criteria, if applicable | Yes | Main outcome measures were “Differences at each timepoint in mean utility values and mean NHS and PSS costs, and differences  in EQ-5D-3L and SF-6D-derived QALYs and cumulative NHS and PSS costs for the one-year follow-up period” (p1507)  CDC diagnostic criteria for SSI (as above).  “age, gender, trial site, Gustilo–Anderson wound grade, presence of diabetes, height, weight, and smoking status” (p1507) were explored as potential confounders. |
| 8: Data sources/measurement  For each variable of interest, give sources of data and details of methods of assessment (measurement). Describe comparability of assessment methods if there is more than one group | Yes | “The data set used for this secondary analysis included all 460 participants in the WOLLF study, regardless of trial allocation” (p1507)  “EuroQol EQ-5D-3L and the 6-Item  Short-Form Health Survey questionnaire (SF-6D), based on participant completion of the EQ-5D-3L and 12-Item Short-Form Health Survey questionnaire (SF-12) measures at baseline and at three, six, nine, and 12 months post-randomization” (p1507) |
| 9: Bias  Describe any efforts to address potential sources of bias | Not clear | OLS regression used to explore the impacts of variables outlined in section 7 (above), but no further information provided.  Original RCT used blinding fo allocation but this was not the focus of the economic study. |
| 10: Study size  Explain how the study size was arrived at | Yes | “The data set used for this secondary analysis included all 460 participants in the WOLLF study” (p1507). |
| 11: Quantitative variables  Explain how quantitative variables were handled in the analyses. If applicable, describe which groupings were chosen and why | Yes | EQ-5D responses converted to utitilit using Dolan UK values (p1507), SF-12 using Brazier and Roberts. QALYs were generated from utility values.  “Economic costs were calculated using participant-completed questionnaires” and “Resource inputs were valued using primary and secondary cost sources” (p1507). |
| Statistical methods  12a: Describe all statistical methods, including those used to control for confounding | Yes | Section Statistical Analysis (p1507). |
| 12b: Describe any methods used to examine subgroups and interactions | Yes | Section Statistical Analysis (p1507).  “age, gender, trial site, Gustilo–Anderson wound grade, presence of diabetes, height, weight, and smoking status” (p1507) |
| 12c: Explain how missing data were addressed | Not clear | Reports 2 patients with missing data (p1508) but no information given as to how these were dealt with in the analysis. |
| 12d: If applicable, explain how loss to follow up was addressed | N/A | Current study is a secondary analysis of data. |
| 12d: If applicable, explain how matching of cases and controls was addressed | Yes | “Two groups of patients were compared: those without, and those with, a diagnosis of deep surgical site infection (SSI), according to the Centres for Disease Control (CDC) definition” (p1507) |
| 12e: Describe any sensitivity analyses | Yes | “a complete case analysis included as a sensitivity analysis” (p1507). |
| Results | | |
| Participants  13a: Report numbers of individuals at each stage of study— e.g. numbers potentially eligible, examined for eligibility, confirmed eligible, included in the study, completing follow-up, and analysed | Yes | Details of patient flow for the RCT given in Parker 2018. Additional information for economic analyses “The study population comprised 460 individuals, 35 of whom had deep SSI, 423 of whom did not, and two patients for whom data were missing.” (p1508). |
| 13b: Give reasons for non-participation at each stage | Yes | As above |
| 13c: Consider use of a flow diagram |  |  |
| Descriptive data  14a: Give characteristics of study participants (eg demographic, clinical, social) and information on exposures and potential confounders | Yes | In Parker 2018, p2283. |
| 14b: Indicate number of participants with missing data for each variable of interest | No | Two missing participant data identified but no further information given. |
| 15: Outcome data  Report numbers in each exposure category, or summary measures of exposure | Yes | “35 of whom had deep SSI, 423 of whom did not” (p1508). |
| Main results  16a: Give unadjusted estimates and, if applicable, confounder-adjusted estimates and their precision (eg, 95% confidence interval). Make clear which confounders were adjusted for and why they were included | Not clear | Economic outcomes in Table i (supplementary materials) only gives utility value stratified according to time since injury. |
| 16b: Report category boundaries when continuous variables were categorized | N/A | Not relevant for economic outcomes |
| 16c: If relevant, consider translating estimates of relative risk into absolute risk for a meaningful time period | N/A | Not relevant for economic outcomes |
| 17: Other analyses  Report other analyses done—e.g. analyses of subgroups and interactions, and sensitivity analyses | Yes | Gives outcomes using utilities from both QoL instruments (EQ-5D and SF-6D). Reports of costs also provided. |
| Discussion | | |
| 18: Key results  Summarise key results with reference to study objectives | Yes | Section Discussion (p1509). |
| 19: Limitations  Discuss limitations of the study, taking into account sources of potential bias or imprecision. Discuss both direction and magnitude of any potential bias | Yes | Acknowledged limitations of small number of cases (n = 35) and follow up period (12 months). (p1509). |
| 20: Interpretation  Give a cautious overall interpretation of results considering objectives, limitations, multiplicity of analyses, results from similar studies, and other relevant evidence | Yes | Section Discussion (p1509). |
| 21: Generalisability  Discuss the generalisability (external validity) of the study results | Yes | Particular focus given to “the multicentre  recruitment of patients, thereby improving the generalizability of the study’s findings across healthcare settings, particularly within the NHS” (p1509). |
| Other information | | |
| 22: Funding  Give the source of funding and the role of the funders for the present study and, if applicable, for the original study on which the present article is based | Yes | NIHR Health Technology Assessment Programme (from the trial registration). |

| STUDY REFERENCE | |  |
| --- | --- | --- |
| ***Totty 2021***  ***Details also taken from trial protocol Totty 2019*** (18) | Enter **Y**es/**P**robably **Y**es/**P**robably **N**o/**N**o/**N**o **I**nformation | Text that helped you make the decision, including page number |
| Domain 1: Risk of bias arising from the randomisation process | | |
| 1.1 Was the allocation sequence random? | Yes | “Consented patients will be randomised to one of the two parallel testing groups (DACC-coated dressing or standard post-operative dressing) by equal randomisation  using the online computerised sealed enveloped method (Sealed Envelope, London, UK)” (p4, Totty 2019). |
| 1.2 Was the allocation sequence concealed until participants were enrolled and assigned to interventions? | Yes | “Randomisation occurred in the theatre after wound closure to prevent performance bias” (p262, Totty 2020). |
| 1.3 Did baseline differences between intervention groups suggest a problem with the randomization process? | No | Statistically significant difference between control and intervention groups for smoking status and diabetes. This is similar to the other RCT in this Review. |
| *Risk-of-bias judgement for Domain 1* | | ***Low*** |
| Domain 2: Risk of bias due to deviations from the intended interventions | | |
| 2.1. Were participants aware of their assigned intervention during the trial? | Probably yes | “Due to the nature of the treatment, a double-blind study is not possible (Leukomed® Sorbact® contains a coloured wound contact layer that is not present on standard dressings)” (p4, Totty 2019). |
| 2.2. Were carers and people delivering the interventions aware of participants' assigned intervention during the trial? | Probably yes | “Due to the nature of the treatment, a double-blind study is not possible (Leukomed® Sorbact® contains a coloured wound contact layer that is not present on standard dressings)” (p4, Totty 2019). |
| 2.3. If Y/PY/NI to 2.1 or 2.2: Were there deviations from the intended intervention that arose because of the trial context? | No | No deviations from intended allocation reported. |
| 2.4 If Y/PY to 2.3: Were these deviations likely to have affected the outcome? | N/A | 2.3 was No |
| 2.5. If Y/PY/NI to 2.4: Were these deviations from intended intervention balanced between groups? | N/A | 2.4 was N/A |
| 2.6 Was an appropriate analysis used to estimate the effect of assignment to intervention? | Yes | “records were not excluded from the primary outcomes analysis” (p262, Totty 2020). |
| 2.7 If N/PN/NI to 2.6: Was there potential for a substantial impact (on the result) of the failure to analyse participants in the group to which they were randomized? | N/A | 2.6 was Yes |
| *Risk-of-bias judgement for Domain 2* | | ***Low*** |
| Domain 3: Risk of bias due to missing outcome data | | |
| 3.1 Were data for this outcome available for all, or nearly all, participants randomized? | Probably yes | “Eight of the 144 records were removed prior to data analysis” (p262, Totty 2020) representing a 6% dropout rate. |
| 3.2 If N/PN/NI to 3.1: Is there evidence that the result was not biased by missing outcome data? | N/A | 3.1 was Yes |
| 3.3 If N/PN to 3.2: Could missingness in the outcome depend on its true value? | N/A | 3.2 was N/A |
| 3.4 If Y/PY/NI to 3.3: Is it likely that missingness in the outcome depended on its true value? | N/A | 3.3 was N/A |
| *Risk-of-bias judgement for Domain 3* | | ***Low*** |
| Domain 4: Risk of bias in measurement of the outcome | | |
| 4.1 Was the method of measuring the outcome inappropriate? | No | Quality of life (outcome of interest for this Review) was EQ-5D-3L. |
| 4.2 Could measurement or ascertainment of the outcome have differed between intervention groups? | Probably no | “All patient data, including patient reported quality of life and clinical outcomes, will be entered into a paper-based case report form (CRF) and anonymised into Microsoft  Excel for further analysis and monitored by the research and development department” (p4, Totty 2019)  “At each study time point, patient-reported HRQoL was recorded using the EQ-5D-3L instrument” (p262, Totty 2020). |
| 4.3 If N/PN/NI to 4.1 and 4.2: Were outcome assessors aware of the intervention received by study participants? | No | “assessor reported outcomes, namely the ASEPSIS scoring of wounds, will be performed by a blinded assessor who will not have  access to the patient notes. Anonymous photos of wounds will be rated by a third blinded assessor to reduce bias and ensure discrepancies in ASEPSIS scores are addressed” (p4, Totty 2019). |
| 4.4 If Y/PY/NI to 4.3: Could assessment of the outcome have been influenced by knowledge of intervention received? | N/A | 4.3 was No |
| 4.5 If Y/PY/NI to 4.4: Is it likely that assessment of the outcome was influenced by knowledge of intervention received? | N/A | 4.4 was N/A |
| *Risk-of-bias judgement for Domain 4* | | ***Low*** |
| Domain 5: Risk of bias in selection of the reported result | | |
| 5.1 Were the data that produced this result analysed in accordance with a pre-specified analysis plan that was finalized before unblinded outcome data were available for analysis? | Yes | Analysis reported in Totty 2020 aligns with that from the protocol in Totty 2019. |
| Is the numerical result being assessed likely to have been selected, on the basis of the results, from... |  |  |
| 5.2. ... multiple eligible outcome measurements (e.g. scales, definitions, time points) within the outcome domain? | No | EQ-5D analysis completed as per convention “Utility values were calculated from the EQ-5D-3L instrument using the UK value set with the R package eq5d v0.0.0” (p263, Totty 2020). |
| 5.3 ... multiple eligible analyses of the data? | No | As above. |
| *Risk-of-bias judgement for Domain 5* | | ***Low*** |
| **Overall risk-of-bias judgement** | | **Low** |

| STROBE criteria | |  |
| --- | --- | --- |
| ***Totty 2021*** | Enter Yes/No/Not clear or NA | Text that helped you make the decision, including page number |
| Title and abstract | | |
| 1a: Indicate the study’s design with a commonly used term in the title or the abstract | No | Not provided. |
| 1b: Provide in the abstract an informative and balanced summary of what was done and what was found | Yes | Section Abstract (p261). |
| Introduction | | |
| 2: Background/rationale  Explain the scientific background and rationale for the investigation being reported | Yes | Section 1 Introduction (p261 – 2). |
| 3: Objectives  State specific objectives, including any prespecified hypotheses | Yes | “The objective of this study was to assess the cost and health-related quality of life impact of SSI, from the perspective of a large teaching hospital in England” (p261). |
| Methods | | |
| 4: Study design  Present key elements of study design early in the paper | Yes | Section 2 Methods (p262 – 3). |
| 5: Setting  Describe the setting, locations, and relevant dates, including periods of recruitment, exposure, follow-up, and data collection | Not clear | “Data from 144 patients treated in a tertiary vascular surgery unit in the United Kingdom” (p262)  No date information provided. |
| Participants  6a: Give the eligibility criteria, and the sources and methods of case ascertainment and control selection. Give the rationale for the choice of cases and controls | Yes | Inclusion/exclusion criteria available from protocol (Totty 2019, p2)  “Study participants were stratified into those who did not experience SSI within 30 days  following surgery (No SSI; n = 107) and those who did (SSI; n = 29).” (p263). |
| 6b: For matched studies, give matching criteria and the number of controls per case | Yes | “Study participants were stratified into those who did not experience SSI within 30 days  following surgery (No SSI; n = 107) and those who did (SSI; n = 29).” (p263). |
| 7: Variables  Clearly define all outcomes, exposures, predictors, potential confounders, and effect modifiers. Give diagnostic criteria, if applicable | Yes | “At each study follow-up time point, patient-reported HRQoL was recorded using the EQ-5D-3L instrument” (p263)  SSI diagnostic criteria from the CDC used (from protocol, Totty 2019).  Variables of “age, sex, body mass index (BMI), smoking status, diabetes, and procedure  type” (p263) explored using a linear regression model. |
| 8: Data sources/measurement  For each variable of interest, give sources of data and details of methods of assessment (measurement). Describe comparability of assessment methods if there is more than one group | Yes | As above (section 5). Data collection for economic outcomes (QoL) was “recorded using the EQ-5D-3L instrument” (p262). |
| 9: Bias  Describe any efforts to address potential sources of bias | Not clear | Regression used to explore the impacts of variables outlined in section 7 (above), but no further information provided.  Original RCT used blinding for allocation but this was not the focus of the economic study. |
| 10: Study size  Explain how the study size was arrived at | Yes | “Data from 144 patients treated in a tertiary vascular surgery unit in the United Kingdom” (p262) |
| 11: Quantitative variables  Explain how quantitative variables were handled in the analyses. If applicable, describe which groupings were chosen and why | Yes | Section 2.1 Data analysis (p262 – 3). |
| Statistical methods  12a: Describe all statistical methods, including those used to control for confounding | Yes | Section 2.1 Data analysis (p262 – 3). |
| 12b: Describe any methods used to examine subgroups and interactions | Yes | Variables of “age, sex, body mass index (BMI), smoking status, diabetes, and procedure  type” (p263) explored using a linear regression model. |
| 12c: Explain how missing data were addressed | Yes | “Eight of the 144 records were removed prior to data analysis” (p262). Missing data may be inferred from the infomraiton provided on the eight removed records. |
| 12d: If applicable, explain how loss to follow up was addressed | N/A | Economic study was a secondary analysis of data. |
| 12d: If applicable, explain how matching of cases and controls was addressed | Yes | “Study participants were stratified into those who did not experience SSI within 30 days  following surgery (No SSI; n = 107) and those who did (SSI; n = 29).” (p263). |
| 12e: Describe any sensitivity analyses | Not clear | None specified the narrative but Table 3 outlines results of sensitivity analysis. Sensititivy analyses reported in relation to length of stay (p266) also. |
| Results | | |
| Participants  13a: Report numbers of individuals at each stage of study— e.g. numbers potentially eligible, examined for eligibility, confirmed eligible, included in the study, completing follow-up, and analysed | Yes | Opening paragraph of section 2.1 Data analysis (p262). |
| 13b: Give reasons for non-participation at each stage | Yes | Opening paragraph of section 2.1 Data analysis (p262). |
| 13c: Consider use of a flow diagram | Yes | Study flow diagram of original RCT available in the protocol publication (Totty 2019, p5). |
| Descriptive data  14a: Give characteristics of study participants (eg demographic, clinical, social) and information on exposures and potential confounders | Yes | Table 2, p 264. |
| 14b: Indicate number of participants with missing data for each variable of interest | Yes | “Complete EQ-5D records were available at baseline for 73 patients who did not develop SSI and 18 patients who did. At day 30, there were 52 and 9 complete records for patients with no SSI and with SSI, respectively.” (p263). |
| 15: Outcome data  Report numbers in each exposure category, or summary measures of exposure | Yes | Table 4, p266. |
| Main results  16a: Give unadjusted estimates and, if applicable, confounder-adjusted estimates and their precision (eg, 95% confidence interval). Make clear which confounders were adjusted for and why they were included | Not clear | Some diagrams and table data refer to “adjusted” results but not enough information is provided to determine how these were derived. |
| 16b: Report category boundaries when continuous variables were categorized | N/A | Not applicable for economic outcomes. |
| 16c: If relevant, consider translating estimates of relative risk into absolute risk for a meaningful time period | N/A | Not applicable for economic outcomes. |
| 17: Other analyses  Report other analyses done—e.g. analyses of subgroups and interactions, and sensitivity analyses | Yes | Length of stay and SSI costs also analysed. |
| Discussion | | |
| 18: Key results  Summarise key results with reference to study objectives | Yes | “In a sample of 136 patients undergoing vascular surgery, 21% developed SSI which was associated with a mean increase in LOS of 9.72 days and an average cost of £3776 per episode” (p266). |
| 19: Limitations  Discuss limitations of the study, taking into account sources of potential bias or imprecision. Discuss both direction and magnitude of any potential bias | Yes | Acknowledged limitations include older average age of sample not being able to provide data on return to work (p266) and SSI treatment costs limited to hospital settings only (p267). |
| 20: Interpretation  Give a cautious overall interpretation of results considering objectives, limitations, multiplicity of analyses, results from similar studies, and other relevant evidence | Yes | Section 4 Discussion. |
| 21: Generalisability  Discuss the generalisability (external validity) of the study results | Yes | Paragraph 3, p267. |
| Other information | | |
| 22: Funding  Give the source of funding and the role of the funders for the present study and, if applicable, for the original study on which the present article is based | Yes | Hull University Teaching Hospitals NHS Trust (from trial registration). |

**TABLE S4**: Quality assessment of economic evaluation

| **Hyldig 2018** | |
| --- | --- |
| ***Study design*** | Enter Yes/No/Not clear or NA and the text that helped you make the decision, including page number |
| The research question is stated | Yes “To evaluate the cost-effectiveness of incisional negative pressure wound therapy…in preventing surgical site infection in obese women after caesarean section” (p619) |
| The economic importance of the research question is stated | Yes, Section Introduction (p619 – 20). |
| The viewpoint(s) of the analysis are clearly stated and justified | Yes “from a Danish healthcare perspective” (p620). |
| The rationale for choosing the alternative programmes or interventions compared is stated | Yes, based on previous RCT work |
| The alternatives being compared are clearly described | Yes, in separate publication (Hyldig et al, 2019. Prophylactic incisional negative pressure wound therapy reduces the risk of surgical site infection after caesarean section in obese women: a pragmatic randomised clinical trial, BJOG, 126(5), pp628 – 35). |
| The form of economic evaluation is stated | Yes “a trial based economic evaluation” and “cost effectiveness analysis” (p619). |
| The form of economic evaluation is justified in relation to the questions addressed | Yes, study aimed to evaluate cost effectiveness of intervention compared to standard dressing. |
| ***Data collection*** |  |
| The source(s) of effectiveness estimates used are stated | Yes “We used individual patient level data from a multicentre RCT” (p620). |
| Details of the design and results of effectiveness study are given (if based on a single study) | Yes, in separate publication (Hyldig et al, 2019. Prophylactic incisional negative pressure wound therapy reduces the risk of surgical site infection after caesarean section in obese women: a pragmatic randomised clinical trial, BJOG, 126(5), pp628 – 35). |
| Details of the method of synthesis or meta-analysis of estimates are given | Yes “The QALY differences between the intervention and control groups were calculated as the mean difference in the EQ-5D index value” (p620). |
| The primary outcome measure(s) for the economic evaluation are clearly stated | Yes “The two outcome measures selected for the cost effectiveness analysis were SSI and QALYs” (p620). |
| Methods to value health states and other benefits are stated | Yes “Data on QALYs were collected using the generic health related quality of life instrument EuroQol EQ-5D-5L” (p620). |
| Details of the subjects from whom valuations were obtained are given | Yes “women with a pre-pregnancy BMI of ≥30 kg/m^2^ who had an emergency or planned CS” (p620). |
| Productivity changes (if included) are reported separately | N/A: not included given healthcare perspective |
| The relevance of productivity changes to the study question is discussed | N/A: not included given healthcare perspective |
| Quantities of resources are reported separately from their unit costs | Yes, Table 1 p623. |
| Methods for the estimation of quantities and unit costs are described | Yes, section Estimation of costs (p620 – 1). |
| Currency and price data are recorded | Yes “Costs were obtained in DKK and converted to Euros at the year 2015 value” (p621). |
| Details of currency of price adjustments for inflation or currency conversion are given | Yes “Costs were obtained in DKK and converted to Euros at the year 2015 value” (p621). |
| Details of any model used are given | Yes, section Cost-effectiveness analysis and Additional analyses on p621-2. |
| The choice of model used and the key parameters on which it is based are justified | Yes, section Cost-effectiveness analysis and Additional analyses on p621-2. |
| ***Analysis and interpretation of results*** |  |
| Time horizon of costs and benefits is stated | Yes “Costs were obtained in DKK and converted to Euros at the year 2015 value” (p621).  “3 month costs were used for the cost assessment. For quality-adjusted life-years…the time horizon was assumed to be 1 year” (p620). |
| The discount rate(s) is stated | N/A “Discounting was not necessary because of the short time-frame of the treatment and benefits” (p620). |
| The choice of rate(s) is justified | N/A: no discounting applied |
| An explanation is given if costs or benefits are not discounted | Yes ““Discounting was not necessary because of the short time-frame of the treatment and benefits” (p620). |
| Details of statistical tests and confidence internals are given for stochastic data | Yes, Tables 1 and 2p, p623-4. |
| The approach to sensitivity analysis is given | Yes “probabilistic sensitivity analysis using Monte Carlo simulation….two “one way” deterministic analyses were conducted” (p621). |
| The choice of variables for sensitivity analysis is justified | Yes, section Additional analyses (p621). |
| The ranges over which the variables are varied are stated | Not clear |
| Relevant alternatives are compared | Yes; dressing and standard dressing and also including comparisons for 3 and 12 month costs, exclusion of outlier, inputting missing hospital costs, excluding missing QALY data and different levels of BMI (from Table 2). |
| Incremental analysis is reported | Yes; ICER per SSI avoided and ICER per QALY gained reported. (p624). |
| Major outcomes are presented in a disaggregated as well as aggregated form | Yes, Table 2. |
| The answer to the study question is given | Yes “economic evaluation also demonstrates that the iNPWT intervention s the dominant strategy per SSI avoided and QALY gained” (p622). |
| Conclusions follow the data reported | Yes |
| Conclusions are accompanied by the appropriate caveats | Yes; limitations acknowledged include not considering time spent on wound dressing and care or post discharge costs. |

**TABLE S5:** Study quality assessment of health state valuation

| STUDY REFERENCE | |  |
| --- | --- | --- |
| **Matza 2019** | Enter Yes/No/Not clear or NA | Text that helped you make the decision, including page number |
| 1. Design | | |
| A1. When exactly were utility measurements done? | No | Information not provided. |
| A2. The timing of utility assessment relative to other questionnaires. | N/A | Only utility assessment carried out. |
| 1. Administration | | |
| B1. How were the utility questions administered (e.g. by interviewer, mailed questionnaires, computer, the Internet, or self-administered via general supervision)? | Yes | By interviewer (p821). |
| If by interview: |  |  |
| 1. What was the interview setting (e.g. face to face, by telephone, or in the hospital)? | Yes | Not stated explicitly but face to face may be inferred from interview procedure narrative (p821). |
| 1. Where were the utility measurements done (city)? | Yes | London and Edinburgh (p820). |
| 1. How were the interviewers trained? | No | Information not provided. |
| 1. How was between-interviewer reliability assessed? | No | Information not provided. |
| 1. What was the interview duration? | No | Information not provided. |
| B2. Response and completion rates | Yes | “Of the 213 participants who attended the interviews, 12 were unable to complete…the analysis included 201 participants” (p822). |
| B3. Efforts (if any) to increase response or completion rates | Not clear | Although “the interviewer made multiple attempts to clarify the task” is mentioned in relation to the 12 incompletions (p822). |
| 1. Health State Descriptions | | |
| C1. Description of health states, if any | Yes | Section Health state development (p820) and health state text provided in supplementary material. |
| C2. How is “perfect health” described? | No | Only reference to “full health” but no description provided as to what this means. |
| C3. How is “worst health” described? | No | Worst health not described. |
| 1. If “dead” was used, how immediate was death? | N/A | Death not used |
| C4. If utility for “own health” was assessed, was own health specified further? | N/A | Utility for own health not used |
| C5. Was the presentation order of the health states randomised? If not, what was the order? | Yes | “participants were randomised to review either the group of joint surgery health states or spine surgery health states first, followed by the other group. Within the joint surgery group and the spine surgery group the health states were presented in a random order” (p821). |
| C6. Were fixed survival durations used for the health states? | Yes | 1 year. |
| C7. How were the survival durations characterised? | N/A | Death not used as end point. |
| C8. How was the fixed survival duration chosen? | N/A | Death not used as end point. |
| C9. Was it made explicit that each duration was followed by death? | N/A | Death not used as end point. |
| C10. Was the subject instructed to assume that survival does not occur with knowledge of the date of death? | N/A | Death not used as end point. |
| C11. Description of treatments, if any (a treatment corresponds to a decision option in a decision tree) | Yes | Within health state texts (from supplementary material). |
| C12. What was the subject instructed to assume, if anything, regarding costs to him or her family about the possible outcomes? | No | Costs to self or family not included in health state text. |
| C13. Was the health state labelled or unlabelled? | Yes | All health states labelled. |
| 1. Description of the Utility Assessment Method | | |
| D1. Which method was chosen (e.g. VAS, TTO, SG or WTP)? | Yes | TTO |
| D2. If more than 1 utility measure was used, was the presentation order randomised? If not, what was the order? | N/A | Only TTO used |
| D3. How was the choice introduced? | Yes | “Along with health states, participants were shown a background information page briefly…offered a choice between living 1 year in the health state being rated or a shorter duration in full health. Choices were alternated between longer and shorter amounts of time in full health, specified in months” (p821). |
| D4. If more than 1 health state was assessed, were they rank ordered first? | Yes | “After the ranking” (p821). |
| 1. If so, was death included in the ordering procedure? | No | No information provided about death in ranking procedure. |
| 1. If there were states worse than dead, how were they handled? | Yes | “Participants were offered a choice between dead (choice1 ) and a 1 year life span (choice 2) beginning with varying amount of time in the health state being rated, followed by full health for the remainder of the 1 year life span” (p821). |
| D5. Were subjects confronted with inconsistencies in their scores, such as a change in health state ordering as inferred from the different utility assessment methods? | Yes | “illogical responses…the interviewer made multiple attempts to clarify the task” (p822). |
| 1. Indifference procedures | | |
| E1. Was matching or choice indifference search procedure used? | Yes | Choice indifference |
| 1. In the case of choice, what were the first 2 choices? | Yes | “Participants were offered a choice between living 1 year in the health state being rated or a shorter duration in full health” (p821). Order of health states were randomised, choices of time alternated starting with 12. |
| 1. Which particular indifference search procedure was used? | N/A | Indifference search procedure not used. |
| 1. What were the criteria for terminating the indifference search procedure? | N/A | Indifference search procedure not used. |
| 1. Did the subject give a final guess within the narrowed down indifference range? | N/A | Indifference search procedure not used. |
| 1. Visual Aids and Software Programs | | |
| F1. Which software program (if any) was used? | N/A | Software not used. |
| 1. If a software programme was used, was it used by the subject alone or was someone present in the start up phase to answer questions or detect misconceptions? | N/A | Software not used. |
| 1. If someone was present, how was he/she trained? | N/A | Software not used. |
| F2. What visual aids, if any, were used? | Yes | “Health states were presented on individual cards, each with a series of bullet point descriptions…To help respondent understand the sequencing of events, a timeline was depicted” (p821) |
| F3. Were there any aspects of the interview controlled by computer? | N/A | No computers used in the valuation interviews. |

**References**

(1) Aboltins C, Dowsey MM, Peel T, Lim WK, Parikh S, Stanley P, et al. Early prosthetic hip joint infection treated with debridement, prosthesis retention and biofilm-active antibiotics: functional outcomes, quality of life and complications. Intern Med J 2013;43(7):810-815.

(2) Cahill JL, Shadbolt B, Scarvell JM, Smith PN. Quality of Life after Infection in Total Joint Replacement. J Orthop Surg (Hong Kong) 2008;16(1):58-65.

(3) Falavigna A, Righesso O, Traynelis VC, Teles AR, da Silva PG. Effect of deep wound infection following lumbar arthrodesis for degenerative disc disease on long-term outcome: a prospective study: clinical article. J Neurosurg Spine 2011 -10;15(4):399-403.

(4) Guirro P, Hinarejos P, Puig-Verdie L, Sánchez-Soler J, Leal-Blanquet J, Torres-Claramunt R, et al. Superficial wound infection does not cause inferior clinical outcome after TKA. Knee Surg Sports Traumatol Arthrosc 2016 -10;24(10):3088-3095.

(5) Haddad S, Núñez-Pereira S, Pigrau C, Rodríguez-Pardo D, Vila-Casademunt A, Alanay A, et al. The impact of deep surgical site infection on surgical outcomes after posterior adult spinal deformity surgery: a matched control study. Eur Spine J 2018 -10;27(10):2518-2528.

(6) Hyldig N, Joergensen JS, Wu C, Bille C, Vinter CA, Sorensen JA, et al. Cost-effectiveness of incisional negative pressure wound therapy compared with standard care after caesarean section in obese women: a trial-based economic evaluation. BJOG 2018 -04;126(5):619-627.

(7) Kuhns BD, Lubelski D, Alvin MD, Taub JS, McGirt MJ, Benzel EC, et al. Cost and quality of life outcome analysis of postoperative infections after subaxial dorsal cervical fusions. J Neurosurg Spine 2015 -04;22(4):381-386.

(8) Matza LS, Kim KJ, Yu H, Belden KA, Chen AF, Kurd M, et al. Health state utilities associated with post-surgical Staphylococcus aureus infections. Eur J Health Econ 2019 -08;20(6):819-827.

(9) Mok JM, Guillaume TJ, Talu U, Berven SH, Deviren V, Kroeber M, et al. Clinical outcome of deep wound infection after instrumented posterior spinal fusion: a matched cohort analysis. Spine (Phila Pa 1976) 2009 -03-15;34(6):578-583.

(10) Parker B, Petrou S, Masters JPM, Achana F, Costa ML. Economic outcomes associated with deep surgical site infection in patients with an open fracture of the lower limb. The Bone & Joint Journal 2018 November 1,;100-B(11):1506-1510.

(11) Pennington Z, Sundar SJ, Lubelski D, Alvin MD, Benzel EC, Mroz TE. Cost and quality of life outcome analysis of postoperative infections after posterior lumbar decompression and fusion. J Clin Neurosci 2019 -10;68:105-110.

(12) Perencevich EN, Sands KE, Cosgrove SE, Guadagnoli E, Meara E, Platt R. Health and economic impact of surgical site infections diagnosed after hospital discharge. Emerg Infect Dis 2003 -02;9(2):196-203.

(13) Petilon JM, Glassman SD, Dimar JR, Carreon LY. Clinical outcomes after lumbar fusion complicated by deep wound infection: a case-control study. Spine (Phila Pa 1976) 2012 -07-15;37(16):1370-1374.

(14) Totty JP, Moss JWE, Barker E, Mealing SJ, Posnett JW, Chetter IC, et al. The impact of surgical site infection on hospitalisation, treatment costs, and health-related quality of life after vascular surgery. Int Wound J 2021 -06;18(3):261-268.

(15) Whitehouse JD, Friedman ND, Kirkland KB, Richardson WJ, Sexton DJ. The impact of surgical-site infections following orthopedic surgery at a community hospital and a university hospital: adverse quality of life, excess length of stay, and extra cost. Infect Control Hosp Epidemiol 2002 -04;23(4):183-189.

(16) Achten J, Parsons NR, Bruce J, Petrou S, Tutton E, Willett K, et al. Protocol for a randomised controlled trial of standard wound management versus negative pressure wound therapy in the treatment of adult patients with an open fracture of the lower limb: UK Wound management of Open Lower Limb Fractures (UK WOLFF). BMJ Open 2015;5(9):e009087.

(17) Costa ML, Achten J, Bruce J, Tutton E, Petrou S, Lamb SE, et al. Effect of Negative Pressure Wound Therapy vs Standard Wound Management on 12-Month Disability Among Adults With Severe Open Fracture of the Lower Limb: The WOLLF Randomized Clinical Trial. JAMA 2018;319(22):2280 - 8.

(18) Totty JP, Harwood AE, Cai PL, Hitchman LH, Smith GE, Chetter IC. Assessing the effectiveness of
dialkylcarbamoylchloride (DACC)-coated post-operative dressings versus standard care in the prevention of surgical site infection in clean or clean-contaminated, vascular surgery (the DRESSINg trial): study
protocol for a pilot feasibility randomised controlled trial. 2019;5(11):1-8.

stylefix

1. Included data from 6 European centres from four different countries [↑](#footnote-ref-1)
2. Charlson comorbidity index [↑](#footnote-ref-2)
